# Machine fault detection model based on MWOA-BiLSTM algorithm

**DOI:** 10.1371/journal.pone.0310133

**Published:** 2024-11-11

**Authors:** Yi-Qiang Xia, Yang Yang

**Affiliations:** College of Science, Liaoning Technical University, Fuxin, China; Torrens University Australia, AUSTRALIA

## Abstract

This paper proposes the Modulated Whale Optimization Algorithm(MWOA), an innovative metaheuristic algorithm derived from the classic WOA and tailored for bionics-inspired optimization. MWOA tackles common optimization problems like local optima and premature convergence using two key methods: shrinking encircling and spiral position updates. In essence, it prevents algorithms from settling for suboptimal solutions too soon, encouraging exploration of a broader solution space before converging, by incorporating cauchy variation and a perturbation term, MWOA achieve optimization over a wide search space. After that, comparisons were conducted between MWOA and seven recently proposed metaheuristics, utilizing the CEC2005 benchmark functions to assess MWOA’s optimization performance. Moreover, the Wilcoxon rank sum test is used to verify the effectiveness of the proposed algorithm. Eventually, MWOA was juxtaposed with the BiLSTM classifier and six other meta-heuristics combined with the BiLSTM classifier. The aim was to affirm that MWOA-BiLSTM outperforms its counterparts, showcasing superior performance across crucial metrics such as accuracy, precision, recall, and F1-Score. The study results unequivocally demonstrate that MWOA showcases exceptional optimization capabilities, adeptly striking a harmonious balance between exploration and exploitation.

## 1. Introduction

In contemporary industrial and commercial settings, maintenance activities transcend mere routine operations; they emerge as pivotal elements in a company’s enduring success. In the realm of mechanical equipment and industrial systems, the management of machine breakdowns directly influences the reliability and cost-effectiveness of production. Notably, in sectors like wind turbine and oil and gas industries, maintenance expenses constitute a substantial portion of the overall costs. For example, reports indicate that the Operation and Maintenance expenses for offshore wind turbines constitute a range of 20% to 35% of the overall revenue generated from electricity production [1], and maintenance expenses within the oil and gas sector may range from 15% to 70% of the overall production costs, reflecting a substantial portion of the total expenses involved in production operations [2]. Furthermore, insufficient maintenance can result in unplanned downtime, adversely affecting not only a company’s core business but also its financial performance directly. In 2013, Amazon experienced a $4 million loss in revenue as a result of just 49 minutes of downtime [3]. This underscores the significance that for major e-commerce entities such as Amazon, even a brief period of downtime can precipitate substantial losses. As per a Ponemon Institute survey, organizations face an average loss of $138,000 for every hour of downtime [4]. This emphasizes that the economic ramifications of downtime are pervasive across diverse industries, exerting a direct and significant influence on a company’s financial position. In conclusion, unplanned downtime of any mechanical equipment or machinery has the potential to undermine or disrupt a company’s core operations, possibly leading to significant penalties and inestimable damage to its reputation [3]. Hence, placing emphasis on maintenance and proactive measures to minimize machine downtime is imperative for enhancing the economic efficiency and sustainability of the company. A well-devised maintenance strategy, coupled with the implementation of preventive maintenance, can aid organizations in averting potential losses and enhancing the stability and reliability of their production systems.

In the face of escalating complexity in manufacturing and equipment, coupled with the limitations of traditional manual troubleshooting methods to address all situations comprehensively [5], modern companies are progressively turning to intelligent solutions to optimize their maintenance strategies [6]. Within the realm of maintenance, machine learning stands out as a pivotal technology facilitating more precise failure prediction [7]. Within the realm of maintenance, machine learning stands out as a pivotal technology facilitating more precise failure prediction. Traditional methods often fall short due to their inability to process and analyze large volumes of data efficiently. Machine learning, however, excels in this area by harnessing extensive historical data and real-time monitoring information. This capability allows systems to learn equipment behavior dynamically. Machine learning models can be trained to identify normal operating modes and potential failure modes, allowing for the timely detection of anomalies and proactive maintenance actions. Malburg et al. [8] evaluated three state-of-the-art target detection systems in order to investigate the suitability of machine learning for detecting artifacts and recognizing faulty situations that require adaptation. Gonzalez-Jimenez et al. [9] introduced a troubleshooting approach leveraging machine learning techniques. This method assists auxiliary maintenance teams in detecting faults specifically within the power connections of induction machines. Tai et al. [10] applying the Hidden Markov Model to the detection of machine failures in process control. In the domain of machine fault detection, support vector machines and long short-term memory in machine learning enjoy widespread usage and preference. SVM excels in handling supervised binary classification problems and stands out for its proficiency in constructing efficient classifiers [11]. Additionally, its capability to accurately classify various classes of failure modes by efficiently identifying the optimal hyperplane renders it applicable to a diverse range of industrial systems. Ghate and Dudul [12] developed a fault detection system for small and medium-sized induction motors utilizing SVM technology. Lee et al. [13] employed SVM for identifying defects caused by shaft misalignment in rotating machinery shafts, while Senanayaka et al. [14] utilized SVM algorithms to promptly detect and classify bearing failures, given their pivotal role in motor and generator malfunctions. Conversely, long short-term memory networks, a category within recurrent neural networks, excel in capturing the complexities of sequential data [15]. LSTMs excel in comprehending and predicting intricate patterns in time series by capturing long-term dependencies within the data. This capability positions them as superior in the domain of machine fault detection. Li et al. [16] developed a pioneering anomaly detection technique for mechanical equipment using SAE-LSTM, allowing for the unsupervised identification of anomalies. Borré et al. [17] tackled the challenge of predicting motor failures by anticipating potential anomalies in the data using CNN-LSTM. In a related context, Han et al. [18] devised a new long short-term memory-based variational autoencoder for fault detection in offshore components on ships.

Although machine learning models like SVMs and LSTMs excel in learning intricate patterns, there are instances where the system’s state may not be fully observable or the data may be affected by noise. In this context, heuristic algorithms emerge as empirical and intuition-based search and optimization techniques that offer a more intuitive and flexible approach to decision-making based on experience and intuition. Their application in machine fault classification and detection arises from the imperative to effectively address optimization problems within complex industrial systems [19]. Within the maintenance domain, heuristic algorithms prove valuable in optimizing maintenance schedules, enhancing equipment life, and reducing costs [20]. By integrating machine learning with heuristic algorithms, we can leverage the strengths of both approaches. Machine learning models can first process large datasets to identify patterns and make initial predictions about potential faults. These predictions can then be refined using heuristic algorithms, which apply domain-specific knowledge and intuitive problem-solving to handle noisy or incomplete data. For example, heuristic algorithms can optimize maintenance schedules based on machine learning predictions, ensuring that maintenance tasks are prioritized effectively and adjusted in real-time to changing conditions.

Additionally, heuristic algorithms can dynamically adjust the hyperparameters of machine learning models, further enhancing their performance. By fine-tuning hyperparameters such as learning rates, regularization terms, and number of hidden layer nodes, heuristic algorithms ensure that machine learning models operate at their optimal capacity. This combination allows for a more adaptive and robust maintenance strategy, ultimately leading to improved equipment life and reduced costs. Through the application of heuristic algorithms, companies can achieve more accurate predictions of potential equipment failures. This, in turn, empowers the development of intelligent, data-driven maintenance plans aimed at minimizing downtime and repair costs [21]. This approach utilizes a large amount of historical data and real-time monitoring information to predict possible failure scenarios. This makes maintenance more predictable and efficient by enabling timely detection of anomalies and preventive maintenance measures in real-time monitoring, minimizing production disruptions and repair costs [22]. By complementing each other with machine learning and heuristic algorithms, a more comprehensive, flexible and intelligent machine fault detection and maintenance management system can be realized. For instance, Cuong-Le et al. [23] proposed a method for damage identification in structural health monitoring and non-destructive damage detection utilizing PSO-SVM. This approach utilizes the structural response under dynamic excitation. Addressing the challenge of low accuracy in the mechanical diagnosis of high voltage circuit breakers, Yang et al. [24] proposed a WOA-SVM-based fault diagnostic. Furthermore, Samanta et al. [25] explored the effectiveness of gear failure detection using GA-SVM.

This paper proposes the Modulated Whale Optimization Algorithm (MWOA), which builds upon the classical WOA in bionics-inspired optimization. MWOA addresses issues of local optimization and premature convergence by modifying the original linear shrinking encircling mechanism and spiral updating position approach. By integrating cauchy variation and a perturbation term, MWOA enhances its performance in navigating complex search spaces, introducing greater complexity and diversity to the previously fixed search method. This algorithm offers a promising solution to optimization problems by providing improved search capabilities and overcoming convergence limitations.

The subsequent sections of this article unfold as follows. In Section 2, a meticulous exposition is provided on the intricate process of constructing the mathematical model for BiLSTM. Section 3 delves into an in-depth elucidation of the modulation mechanism in MWOA. Moving forward, Section 4 meticulously evaluates the efficacy of MWOA on the CEC2005 benchmark function, juxtaposing its performance against seven prominent nature-inspiredmetaheuristic algorithms and then performs the Wilcoxon rank sum test. Furthermore, Section 5 meticulously tests and compares the performance of the MWOA-BiLSTM model against six other models for machine fault detection. Finally, Section 6 encapsulates the findings and proffers insights into future avenues of research.

## 2. Related work

In this section, we present the principles of Long Short-Term Memory (LSTM) neural networks and Bidirectional Long Short-Term Memory (BiLSTM) neural networks, as well as the Whale Optimization Algorithm (WOA). By understanding these foundational concepts, we lay the groundwork for improving metaheuristic algorithms and their integration with machine learning models in subsequent discussions.

### 2.1 LSTM principle of neural network

In the realm of machine learning, the issue of gradient vanishing poses a pervasive challenge [26], particularly pronounced when handling lengthy sequential data. Traditional Recurrent Neural Networks encounter difficulties over multiple iterations as the gradient information may swiftly approach zero (resulting in gradient vanishing) or experience rapid escalation (leading to gradient explosion) due to successive multiplication operations [27]. This phenomenon significantly impedes the network’s ability to effectively learn dependencies in long time-series sequences.

**Fig 1:**
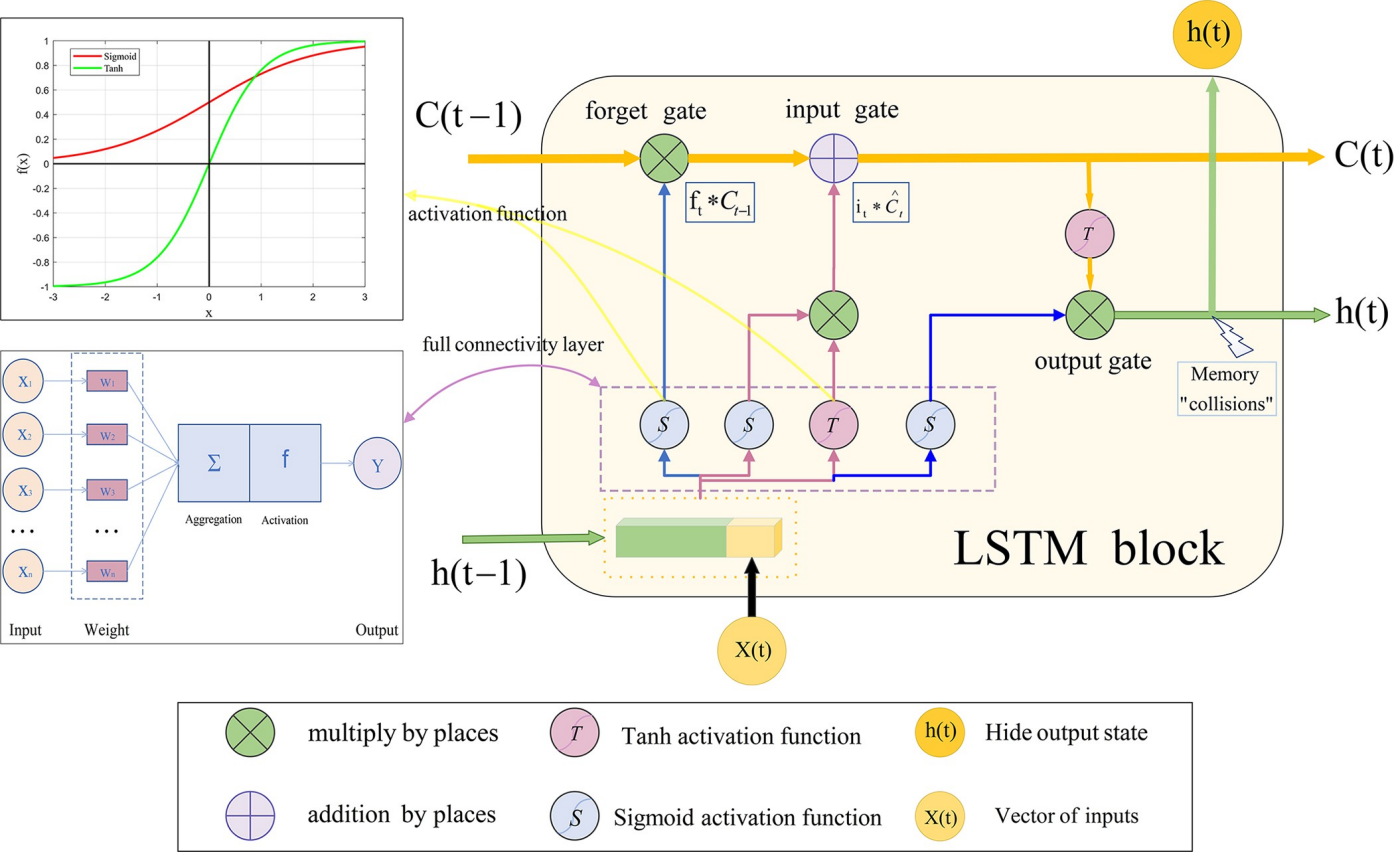
LSTM regulation principle.

To tackle this challenge, Hochreiter and Schmidhuber proposed the concept of memory units in 1997 as a solution to the issues of inadequate gradient and diminishing error backpropagation in traditional RNN training [28]. The essence of the memory unit lies in preserving a persistent state [29], empowering the network to adeptly retain crucial information when handling extensive sequences [30]. This design attains meticulous control over the memory unit’s state by incorporating three gates to modulate the flow of information. Refer to Fig 1 for a comprehensive elucidation of the intricate details within.

The fundamental concept behind LSTM is to employ memory cells as autonomous activation functions and constant functions with steadfast weights connected to themselves [30]. The crucial aspect lies in the stability introduced by these fixed weights, preventing the gradient from either vanishing or exploding during back-propagation through memory cell errors [31]. This innovative architecture significantly improves LSTM’s capability to capture temporal dependencies within extensive sequential data [32], proficiently addressing the issue of vanishing gradients and establishing itself as a potent tool for handling intricate temporal tasks.

The forget gate regulates the extent to which old information will be discarded. The formula is as follows Eqs (1) and (2).

$$f_{t}=\sigma (W_{f}*[h_{t-1},x_{t}]+b_{f})$$ (1)

$$C_{t}=f_{t}*C_{t-1}+i_{t}*\overset{\wedge }{C_{t}}$$ (2)

where *σ* represents the sigmoid function, *f*_*t*_ varies between 0 and 1, and *C*_*t*_ also falls within the range of 0 to 1. *W*_*f*_ denotes the weight of the forget gate, while *x*_*t*_ stands for the input of the current layer at time *t*, and *h*_*t*−1_ represents the output at the previous time step.

The input gate is tasked with determining the amount of new information to be incorporated into the memory cell. Eq (3) and (4) delineate the mathematical formulations in question.

$$i_{t}=\sigma (W_{i}*[h_{t-1},x_{t}]+b_{i})$$ (3)

$$\overset{\wedge }{C_{t}}=\tanh(W_{c}*[h_{t-1},x_{t}]+b_{c})$$ (4)

where i_*t*_ ranges between 0 and 1, *W*_*i*_ represents the weight of the input gate, *b*_*i*_ signifies the input gate bias, *W*_*c*_ denotes the weight of the candidate gate, and *b*_*c*_ stands for the bias of the candidate gate.

By employing the sigmoid activation function, the inputs and previous hidden states undergo weighting and summation, producing a gating signal within the (0,1) range. This signal plays a pivotal role in determining the significance of each element. Concurrently, the input and previous hidden states are subjected to the tanh activation function, yielding a new information vector. The input gates then perform element-wise multiplication on the information, thereby defining the new information that will be integrated into the memory cell.

The output gate generates the final memory cell state by blending the Sigmoid with the tanh activation function. At time *t*, the input to the output gate consists of the outputs *h*_*t*−1_ and input *x*_*t*_, while the output *o*_*t*_ is determined by computing Eqs (5) and (6) according to the following formula.


$$o_{t}=\sigma (W_{o}*[h_{t-1},x_{t}]+b_{o})$$ (5)

$$h_{t}=o_{t}*\tanh(C_{t})$$ (6)

The input and the previous hidden state undergo processing via the tanh activation function, resulting in a new information vector. The output gate performs element-wise multiplication on the aforementioned pieces of information to derive the ultimate memory cell state, which is subsequently transmitted to the next layer of the network.

### 2.2 BiLSTM principle of neural network

**Fig 2:**
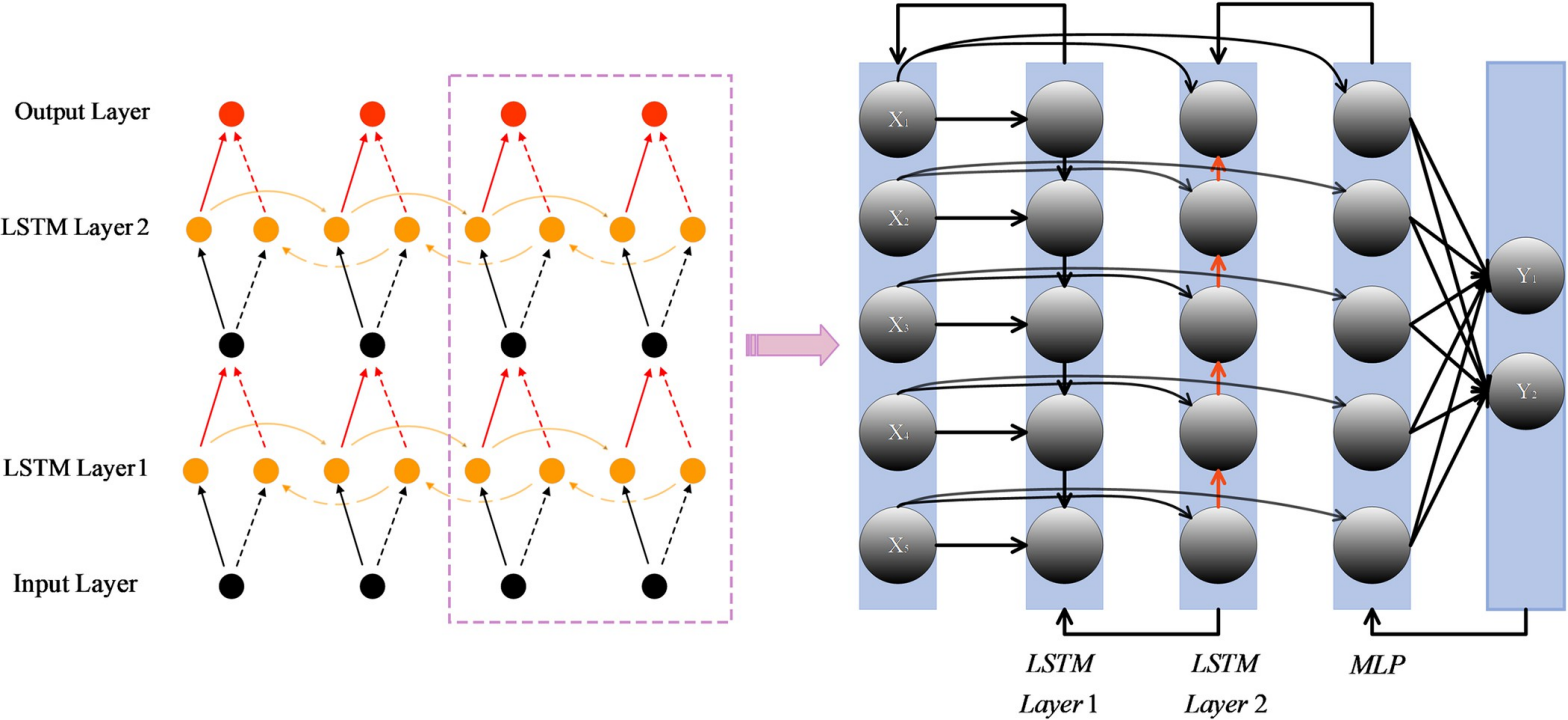
BiLSTM internal network update method.

Deep bidirectional LSTM networks (BiLSTM) [33] present a refinement of traditional LSTMs (illustrated in Fig 2). Unlike the standard training paradigm, which advances strictly from inputs to outputs [34], BiLSTM distinguishes itself by undergoing bidirectional training—simultaneously from both inputs to outputs and from outputs to inputs [35]. In essence, it assimilates training information in both directions, rendering BiLSTM more potent in capturing temporal relationships and comprehensively understanding context. In terms of structure, LSTM exhibits a symmetric unidirectional configuration where the parameters remain consistent across all time steps [36]. In contrast, BiLSTM adopts an asymmetric structure, featuring independent forward and backward parameters. This asymmetry enhances the model’s complexity.

Another crucial distinction lies in parameter updating and hidden states. LSTM conducts parameter updating through both forward and backward propagation [37], with the gradient propagating from moment t to t-1. In contrast, BiLSTM executes both forward and backward propagation in both directions simultaneously, allowing the gradient to propagate through both directions concurrently. This concurrent propagation enhances the efficiency of capturing long-range dependencies [38]. Furthermore, LSTM features a single hidden state, encapsulating the forward information of the sequence. In contrast, BiLSTM incorporates two hidden states, representing both forward ($h_{t}^{\left(f\right)}$) and backward ($h_{t}^{\left(b\right)}$) information. The ultimate output of BiLSTM involves combining these two hidden states to create a more comprehensive representation, denoted as $h_{t}^{\left(bi\right)}=[h_{t}^{\left(f\right)},h_{t}^{\left(b\right)}]$.

After recognizing the potent sequence modeling capabilities of BiLSTM, we delve into its practical applications. BiLSTM has gained widespread usage in the processing of time-series data [39], owing to its exceptional performance in handling complex sequence relationships. Specifically, within the realm of machine fault detection, Yahyaoui et al. [40] proposed a KPCA-based BiLSTM approach for efficiently detecting and diagnosing faults in power converters within wind turbine systems. Jiahao et al. [41] introduced SVM-BiLSTM, a deep learning-based fault detection method tailored for IoT systems at gas stations. Bharatheedasan et al. [42] introduced an approach based on CNN-BiLSTM for diagnosing rolling bearing faults.

However, it’s notable that most of these applications incorporating BiLSTM are rooted in machine learning methods [43], and there exists a scarcity of instances where BiLSTM is combined with heuristic algorithms for machine fault detection and classification. Zhang et al. [44] proposed a novel method for predicting ship motion attitude, leveraging an adaptive dynamic particle swarm optimization algorithm in combination with BiLSTM. Zhen et al. [45] introduced a GA-based improved Bi-LSTM for microgrid photovoltaic power prediction. Heuristic algorithms, known for their global search capabilities and extensive exploration of parameter space, prove valuable in finding optimal model configurations. This is particularly crucial for parameter-heavy BiLSTM models.

### 2.3 Whale optimization algorithm

**Fig 3:**
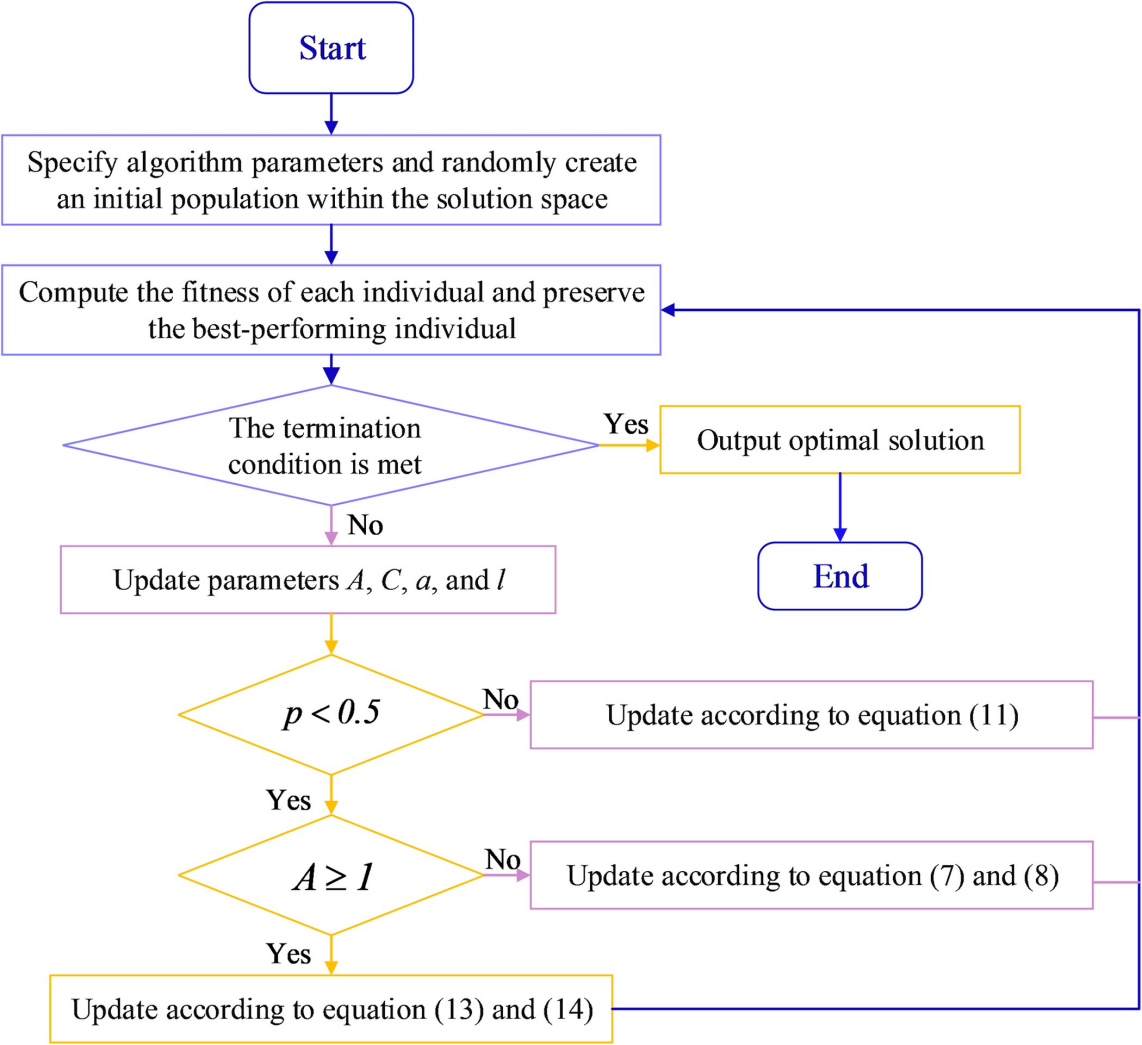
WOA process.

WOA represents a collective intelligence algorithm crafted to tackle continuous optimization issues [46]. Empirical evidence supports the assertion that this algorithm demonstrates superior or comparable performance when benchmarked against several existing algorithmic techniques [47]. The inspiration behind WOA can be traced to the fascinating hunting behavior observed in humpback whales [47]. This choice of modeling, derived from the natural world, underscores the algorithm’s effectiveness in navigating and optimizing complex solution spaces [48]. In the context of WOA, every solution is metaphorically represented as a whale. Within this conceptualization, each whale, or solution, endeavors to explore a novel location within the search space, using the best-performing element in the group as a reference. This mimicry of the natural behavior of whales reflects the algorithm’s strategy of dynamically adjusting and refining individual solutions based on the success of the collective [49]. The utilization of such a bio-inspired approach enhances the algorithm’s adaptability and efficiency in navigating the optimization landscape. The WOA process is shown in Fig 3. The fundamental WOA comprises three primary procedures.

1. Surrounding and encompassing prey.
2. Utilizing bubble-net tactics during the exploitation stage.
3. Hunting for prey during the exploration phase.

#### 2.3.1 Surrounding and encompassing prey

Humpback whales demonstrate an extraordinary skill in locating and surrounding their prey. Similarly, the WOA operates under the assumption that, like whales discerning the best prey locations, the target solution within the search space is either the optimal candidate or closely located to the optimum. Upon identifying the most promising search agent and understanding its characteristics, the subsequent phase entails other search agents adjusting their positions towards this optimal agent. This adaptive behavior is mathematically expressed through Eqs (7) and (8), as detailed in the literature [47]:

$$\vec{D}=\left|\vec{C}\cdot \vec{X^{*}}(t)-\vec{X}(t\right)|$$ (7)

$$\vec{X}(t+1)=\vec{X^{*}}(t)-\vec{A}\cdot \vec{D}$$ (8)

where *t* denotes the present iteration, vectors $\vec{A}$ and $\vec{C}$ denote coefficients. $\vec{X^{*}}$ represents the positional vector of the most optimal solution achieved thus far, $\vec{X}$ signifies the vector denoting position, || denotes the magnitude. An important observation is that variable $\vec{X^{*}}$ needs to be updated in each iteration whenever a better solution is found. This emphasizes the continuous refinement of $\vec{X^{*}}$ to incorporate any improvements found during the iterative process. Vectors $\vec{A}$ and $\vec{C}$ are computed according to Eqs (9) and (10) respectively.


$$\vec{A}=2\vec{a}\cdot \vec{r}-\vec{a}$$ (9)

$$\vec{C}=2*\vec{r}$$ (10)

During the iterative process, as it progresses, the parameter $\vec{a}$ transitions from an exploration phase to an exploitation phase. Meanwhile, $\vec{r}$ is represented by a random vector within the interval [0, 1].

#### 2.3.2 Bubble-net attacking strategy

Two different methodologies are used to develop a mathematical model for the whales. These approaches are described as follows.

1. Shrinking encircling mechanism:
  To instill this behavior, the parameter $\vec{a}$ in Eq (9) undergoes a reduction. It’s important to emphasize that the variability range of $\vec{A}$ is also constrained by $\vec{a}$. To put it more straightforwardly, $\vec{A}$ denotes a random value within the range of [-a, a], while $\vec{a}$ gradually diminishes from [0,2] with each iteration. When randomly assigning values to $\vec{A}$ from the interval [-1, 1], the recalculated position of a search agent falls between its initial position and the location of the current optimal agent.
2. Spiral updating position:
  In crafting this behavior, the researchers computed the distance between the current whale position and the prey. Following this distance determination, they devised a spiral equation, illustrated in Eq (11) [47], to replicate the whale’s spiral movement from its existing location to the prey position.

X→(t+1)=D→'*ebl*cos(2πl)+X*→(t) (11)

In this context, D→'=|X*→(t)−X→(t)| represents the distance between the whale and its prey, which is the best solution obtained up to the current moment, while *b* signifies a constant determining the logarithmic spiral shape. The *l* represents a randomly generated number within the interval [-1, 1].

When simulating the movements of humpback whales around prey, a notable observation is the simultaneous utilization of a shrinking circling technique and a spiral path toward the prey. Additionally, the probability of whales transitioning between these two behaviors is set at 50%, and this transition is mathematically modeled through Eq (12) [47]:

X→(t+1)={X*→(t)−A→⋅D→ifp<0.5D→'*ebl*cos(2πl)+X*→(t)ifp≥0.5 (12)

where *p* is a randomly generated number within the range of [0, 1].

#### 2.3.3 Hunting for prey

An alternative approach, which hinges on the manipulation of the $\vec{A}$ vector’s variation, can also be utilized during the exploration phase when hunting for prey. Essentially, humpback whales engage in random searches relative to each other’s positions. In this scenario, the $\vec{A}$ vector, characterized by random values exceeding 1 or falling below -1, directs a search agent to move significantly away from a designated reference whale. Unlike the exploitation phase, where the best-performing agent dictates movement, the exploration phase involves updating a search agent’s position based on a randomly selected peer rather than the top-performing one. This mechanism, along with the condition |A| > 1, accentuates exploration, enabling the WOA to conduct a comprehensive exploratory quest on a global scale. The corresponding formula is as follows Eqs (13) and (14).


$$\vec{D}=|\vec{C}\cdot \vec{X_{\mathrm{rand}}}-\vec{X}|$$ (13)

$$\vec{X}(t+1)=\vec{X_{\mathrm{rand}}}-\vec{A}\cdot \vec{D}$$ (14)

The variable $\vec{X_{\mathrm{rand}}}$ denotes a randomly determined position, signifying the location of the whale that has been selected randomly from the pool of available whales. This random selection process contributes to the diversity and exploration aspects of the algorithm.

## 3. Improved whale optimization algorithm

In this section, we will propose two improvement strategies aimed at solving the problems of local optimality and slow convergence in the whale bubble-net attacking strategy. By introducing these improvements, we will enhance the performance of the algorithm in complex optimization problems and effectively avoid falling into local optimal solutions. Meanwhile, we propose a Modulated Whale Optimization Algorithm (MWOA) that combines these strategies to enhance the global search capability and speed up the convergence rate, thus improving the overall optimization results.

### 3.1 Insufficiency of the algorithms

The WOA algorithm stands out among optimization techniques by drawing inspiration from the sophisticated hunting strategies employed by humpback whales [50]. It intricately mimics the collaborative hunting behaviors of these marine mammals, offering a unique approach to navigating and optimizing solution spaces [51]. In contrast to other algorithms that may derive inspiration from various sources with distinct search strategies and communication methods, WOA distinctly models the foraging conduct of humpback whales during their pursuit of prey. Much like the GWO, WOA incorporates specific mechanisms for information exchange and knowledge sharing among its search agents [52]. However, WOA is not without its limitations. Primarily, it shows sensitivity to the initial solution selection, where the quality of the chosen starting point significantly influences the algorithm’s ultimate optimization performance. Furthermore, the convergence speed of WOA may exhibit variability, potentially leading to instability and the emergence of locally optimal solutions.

Moreover, when confronted with high-dimensional problems, WOA encounters challenges. The simulation of whale behavior may necessitate adaptation to effectively explore spaces with numerous dimensions. While WOA boasts distinctive characteristics and advantages, it is imperative to recognize and address these limitations in practical applications. As a result, it remains crucial to carefully choose an optimization algorithm that suits the particular problems and needs at hand.

### 3.2 The Modulated whale optimization algorithm

WOA strategically employs parameters to find a delicate equilibrium between exploration and exploitation. Nevertheless, it encounters formidable challenges, including the persistence of suboptimal solutions and premature convergence, hindering the overall advancement of the algorithm. Within the WOA, a group of whales assumes the pivotal role of guiding the search, with specific whales designated as leader whales presumed to occupy optimal positions for prey consumption. In response to the inherent limitations of the WOA, we introduce an enhanced variant named the MWOA. MWOA incorporates a modulation mechanism into the whale guidance process, aiming to mitigate issues related to premature convergence and the stagnation of suboptimal solutions. In MWOA, the three primary phases undergo a redefinition, and modulation is strategically employed to dynamically adjust their influence throughout the optimization process.

By augmenting the original WOA with modulation strategies, MWOA endeavors to surmount algorithmic challenges and enhance convergence speed. This refinement not only preserves the fundamental essence of WOA but also introduces novel features to bolster the exploration-exploitation balance, effectively addressing identified shortcomings in the original algorithm.

Initially, enhancing parameter ***a*** involves transitioning from linear adjustments to nonlinear modifications, aiming to strike ***a*** harmonious equilibrium between exploration and exploitation. Parameter ***a***’s determination follows the Eq (15).


$$a=\max(a_{\min},\min(a_{\max},a_{0}*e^{-kt}))$$ (15)

**Fig 4:**
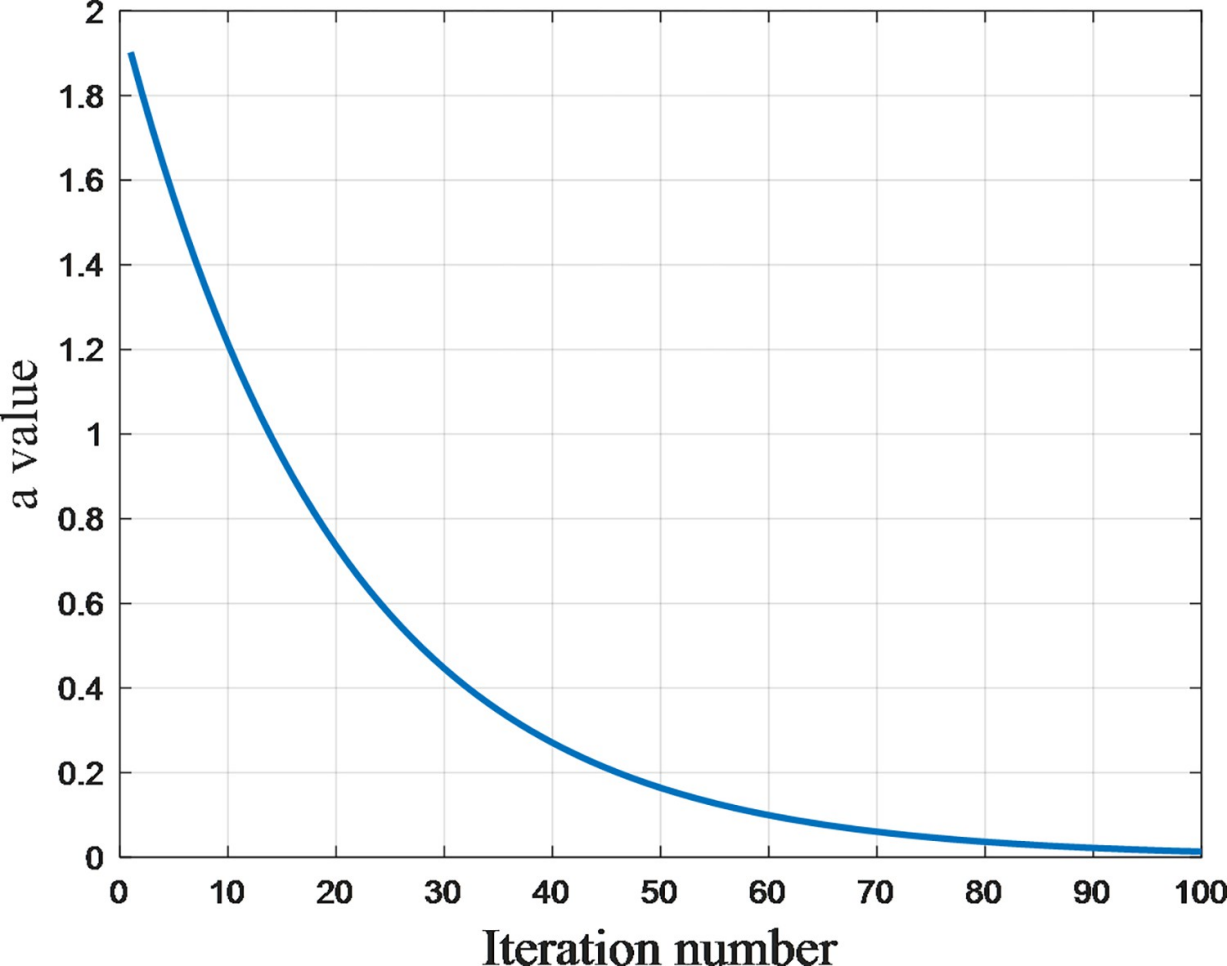
The variation of a with t (take T = 100 for example).

This formula indicates that the parameter *a* experiences exponential decay as the number of iterations increases. This implies that initially, *a* decreases rapidly but gradually slows down as iterations progress. The use of exponential decay offers a notable advantage over linear decay, providing greater flexibility in parameter tuning. More precisely, it enables rapid exploration of the search space during initial iterations, followed by a more focused approach to local refinement in later iterations. This attribute is advantageous in optimization algorithms as it allows for a delicate equilibrium between the exploration of broader solutions and the exploitation of more localized ones. The variation of a with t is shown in Fig 4.

In the context of exponential decay, it becomes essential to set constraints on the range of *a* to maintain algorithmic stability and reasonability. Imposing limits, denoted as *a*_min_ for the minimum and *a*_max_ for the maximum, ensures that *a* stays within a reasonable range [*a*_min_, *a*_max_]. These constraints play a crucial role in preventing *a* from becoming excessively small or large, thereby upholding the stability of the algorithm. The judicious application of range restrictions contributes to a more controlled and effective optimization process.

#### 3.2.1 Adaptive control parameters

The introduction of control-type parameters α and β serves the purpose of addressing challenges faced by the original algorithm when confronted with diverse search space features. Without these parameters, the spiral motion’s shape could become overly rigid during certain iteration phases. This inflexibility in the spiral shape constrains the algorithm’s performance across various problem spaces, particularly in complex, dynamic, or multimodal search spaces.The consequence of this rigidity is a potential bias of the algorithm towards extensive exploration in some stages and excessive focus on local optimization in others. These biases result in a search procedure that lacks the required adaptability to accommodate the changing attributes of the search space across different iteration counts. Integrating control parameters *α* and *β* introduces flexibility, empowering the algorithm to dynamically alter the spiral motion’s configuration as iterations progress.

The adaptability inherent in this feature promotes a refined equilibrium between exploration and exploitation, thereby improving the algorithm’s capacity to navigate intricate and dynamic problem landscapes. Ultimately, the refined algorithm with these control-type parameters exhibits improved flexibility and adaptability, mitigating the limitations observed in the original approach. Following the inclusion of adaptive control parameters, the model is represented by Eq(16).

X→(t+1)=D→'*e(−β*t)*cos(α*t)+X*→(t) (16)

where t denotes the current number of iterations, α is an adaptive parameter controlling the helix period, β is an adaptive parameter controlling the helix shape.

The parameterization of the number of iterations, denoted as *t*, plays a crucial role in shaping the spiral motion, inducing a gradual reduction during the optimization process. This deliberate adjustment aids the algorithm in conducting an extensive search in the initial stages, progressively honing in on more accurate regions in the later stages.

The incorporation of the *α* and *β* parameters facilitates the dynamic adjustment of the spiral motion’s shape with increasing iterations. This adaptability enhances the algorithm’s flexibility, enabling it to effectively respond to diverse search space features. Introducing the α parameter allows for the modulation of the spiral motion’s frequency. A smaller α results in a broader spiral shape, emphasizing global search, while a larger α leads to a more compact spiral, enhancing focus on detailed local search. The inclusion of the *e*^(−β*t)^ term enables the utilization of larger spiral amplitudes in the early iterations, promoting extensive exploration. As the iterations progress, the amplitude gradually diminishes, fostering a more refined local search.

This refinement significantly accelerates the algorithm’s convergence speed, particularly in complex search spaces. The dynamic adjustment of the spiral motion expedites the convergence towards the optimal solution across the entire search space. Showcasing the algorithm’s effectiveness in navigating intricate optimization landscapes.

#### 3.2.2 Cauchy perturbation

By incorporating cauchy variation and a perturbation term, the algorithm introduces increased diversity in each iteration [53]. This strategic improvement helps prevent the algorithm from getting stuck in local optimal solutions, thus enhancing its performance in complex search environments. The utilization of cauchy variation and the perturbation term enhances the algorithm’s exploratory nature, facilitating a broader exploration throughout the entire search space. The elongated tail of the cauchy distribution is particularly beneficial, allowing the generation of samples at more distant locations. This elongation effectively increases the algorithm’s likelihood of traversing the entire search space. The introduction of the cauchy variant promotes a higher probability of exploring beyond the current localized region in the search space, contributing to a more comprehensive exploration across the complete solution space. In summary, the incorporation of cauchy variation and the perturbation term imparts a vital exploratory element to the algorithm, preventing it from becoming ensnared in local optima. and enabling a more comprehensive exploration of complex search spaces. The cauchy variational perturbation is shown in Eq(17).

$$\vec{Cauchy}=\frac{1}{\pi \gamma [1+(\frac{\vec{X^{*}}-\vec{X}}{\gamma })]}$$ (17)

where $\vec{X^{*}}$ stands for the vector representing the currently optimal solution, while $\vec{X}$ refers to the vector indicating the present position. And *γ* is the scale parameter of the cauchy distribution.

In instances where the disparity between the currently optimal solution $\vec{X^{*}}$ and the present position $\vec{X}$ is substantial, the denominator of the cauchy distribution becomes larger. Consequently, this leads to a smaller perturbation term, diminishing the impact of random perturbations. This deliberate adjustment serves to mitigate random perturbations during the global search phase, thereby preventing premature convergence to a local optimal solution. This modification transforms the spiral updating position phase into Eq (18).

X→(t+1)={X*→(t)−A→⋅D→+G→⋅Cauchy→ifp<0.618D→'*e(−β*t)*cos(α*t)+X*→(t)+G→⋅Cauchy→ifp≥0.618 (18)

where *p* is a random number in the range [0, 1]. $\vec{G}$ and $\vec{Cauchy}$ are constant vectors controlling the cauchy variation and random vectors obeying the cauchy distribution, respectively. The pseudocode of MWOA is shown in Table 1.

**Table 1:** Pseudo-code of MWOA algorithm.

*Algorithm 1*. *Pseudo-code of MWOA algorithm*
*1*. *Set the initial population of whales as X*_*i*_ *(i = 1*, *2*,.* *.* *., *n)*.
*2*. *Evaluate the performance of each search agent by computing their fitness*.
*3*. X^*^ *= the top-performing search agent*.
*4*. ***while*** *(t < the upper limit for iterations)*:
*5*. ***for*** *every individual search agent*:
*6*. *Update parameters a*, *A*, *C*, *l*, *and p with modifications*
*7*. *a = max(a*_*min*_, *min(a*_*max*_, *a*_*0*_ * e ^*-kt*^*))*
*8*. *A*, *C*, *l*, *p = update parameters with modifications()*
*9*. ***if1*** *p < 0*.*618*
*10*. ***if2*** *abs(A) < 1*
*11*. *Update position Eq*.*7(present search agent)*
*12*. ***else if2*** *abs(A) ≥ 1*
*13*. *X*_*rand*_ *= select random search agent()*
*14*. *Update position modified Eq*.*14(present search agent*, *X*_*rand*_*)*
*15*. ***end if2***
*16*. ***elif1*** *p ≥ 0.618*
*17*. *Update position modified Eq*.*16(present search agent*, X^*^)
*18*. ***end if1***
*19*. ***end for***
*20*. *Verify if any search agent exceeds the specified search space boundaries and make necessary adjustments*.
*21*. *Evaluate the performance score for each search agent*.
*22*. Revise X^*^ *if an improved solution is identified*.
*23*. *t = t+1*
*24*. ***end while***
*25*. return X^*^

## 4. Simulation results and analysis

To assess the performance of the MWOA, a series of experiments were conducted across 23 benchmark functions widely utilized in previous research [54]. These functions have been categorically classified into unimodal functions, characterized by a limited number of local minima, and multimodal functions, known for possessing numerous local minima (refer to Tables 2–4). The breakdown of these functions is as follows.

1. Unimodal Benchmark Functions (F1~F7). These functions have one and only one global best advantage, so they are mainly used to test the local search ability and convergence efficiency of optimization algorithms. The goal of unimodal function is to quickly find the global optimal solution, which is suitable for evaluating the convergence speed and optimization accuracy of the algorithm. Such functions are generally simple and easy to compute.
2. Multimodal Benchmark Functions (F8~F13). The multimodal function F8~F13 has many local optimal advantages, so it is easy to fall into the local optimal situation in the optimization process. This kind of function is mainly used to test the global search ability of the algorithm, that is, whether it can jump out of the local optimal effectively and find the global optimal solution. Such functions are designed to be more complex, which can simulate the complex search space in the real problem and challenge the global optimization ability of the algorithm.
3. Fixed-Dimension Multimodal Benchmark Functions (F14~F23). Such functions have multimodal characteristics, but their dimensions are fixed and usually lower. These functions are used to test the optimization ability and stability of the algorithm in a specific dimension. Multi-modal functions with fixed dimensions are usually designed to be more complex, containing more local optima and complex function structures, which are suitable for testing the performance of algorithms in specific scenarios, especially in the case of limited dimensions in practical applications.

**Table 2:** Description of unimodal benchmark functions.

Benchmark Functions	Dim	Search space	*f*_*min*_
$F_{1}(x)=\sum_{i=1}^{n}x_{i}^{2}$	30	[-100,100]^D^	0
$F_{2}(x)=\sum_{i=1}^{n}\left|x_{i}\right|+\prod_{i=1}^{n}\left|x_{i}\right|$	30	[-10,10]^D^	0
$F_{3}(x)={\sum_{i=1}^{n}\left(\sum_{j‐1}^{i}x_{j}\right)}^{2}$	30	[-100,100]^D^	0
$F_{4}(x)=max_{i}\{|x_{i}|,1\leq i\leq n\}$	30	[-100,100]^D^	0
$F_{5}(x)=\sum_{i=1}^{n‐1}\left[100{\left(x_{i+1}‐x_{i}^{2}\right)}^{2}+{\left(x_{i}‐1\right)}^{2}\right]$	30	[-30,30]^D^	0
$F_{6}(x)={\sum_{i=1}^{n}\left([x_{i}+0.5]\right)}^{2}$	30	[-100,100]^D^	0
$F_{7}(x)=\sum_{i=1}^{n}ix_{i}^{4}+random[0,1)$	30	[-1.28,1.28]^D^	0

**Table 3:** Description of multimodal benchmark functions.

Benchmark Functions	Dim	Search space	*f*_*min*_
$F_{8}(x)=\sum_{i=1}^{n}‐x_{i}sin(\sqrt{\left|x_{i}\right|})$	30	[-500,500]^D^	-12569.5
$F_{9}(x)=\sum_{i=1}^{n}\left[x_{i}^{2}‐10cos(2\pi x_{i})+10\right]$	30	[-5.12,5.12]^D^	0
$F_{10}(x)=‐20exp(‐0.2\sqrt{(1/n)\sum_{i=1}^{n}x_{i}^{2}})‐exp((1/n)\sum_{i=1}^{n}cos(2\pi x_{i}))+20+e$	30	[-32,32]^D^	0
$F_{11}(x)=(1/4000)\sum_{i=1}^{n}x_{i}^{2}‐\prod_{i=1}^{n}cos(x_{i}/\sqrt{i})+1$	30	[-600,600]^D^	0
$\begin{array}{l} F_{12}(x)=(\pi /n)\{10\sin^{2}(\pi y_{1})+\sum_{i=1}^{n‐1}(y_{i}‐1)2[1+10\sin^{2}(\pi y_{i+1})]+(y_{n}‐1)2\}\\ \;+\sum_{i=1}^{n}u(x_{i},10,100,4) \end{array}$ $y_{i}=1+\frac{x_{i}+1}{4},u(x_{i},a,k,m)=\{\begin{array}{l} k(x_{i}‐a{)}^{m}\;x_{i}>a\\ \;0\;‐a<x_{i}<a\\ k(‐x_{i}‐a{)}^{m}\;x_{i}<‐a \end{array}$	30	[-50,50]^D^	0
$\begin{array}{l} F_{13}(x)=0.1\{\sin^{2}(3\pi x_{1})+\sum_{i=1}^{n‐1}(x_{i}‐1{)}^{2}[1+\sin^{2}(3\pi x_{i+1})]\\ \;+(x_{n}‐1{)}^{2}[1+\sin^{2}(2\pi x_{n})]\}+\sum_{i=1}^{n}u(x_{i},5,100,4) \end{array}$	30	[-50,50]^D^	0

**Table 4:** Description of fixed-dimenstion multimodal benchmark functions.

Benchmark Functions	Dim	Search space	*f*_*min*_
$F_{14}(x)={\left[1/500+\sum_{j=1}^{25}\left(1/j+\sum_{i=1}^{2}{\left(x_{i}‐a_{ij}\right)}^{6}\right)\right]}^{‐1}$	2	[-62.536,62.536]^D^	0.998004
$F_{15}(x)={\sum_{i=1}^{11}\left[a_{i}‐(x_{1}(b_{i}^{2}+b_{i}x_{2})/b_{i}^{2}+b_{i}x_{3}+x_{4})\right]}^{2}$	4	[-5,5]^D^	0.000308
$F_{16}(x)=4x_{1}^{2}‐2.1x_{1}^{4}+\frac{1}{3}x_{1}^{6}+x_{1}x_{2}‐4x_{2}^{2}+4x_{2}^{4}$	2	[-5,5]^D^	-1.031629
$F_{17}(x)={\left(x_{2}‐\frac{5.1}{4\pi^{2}}x_{1}^{2}+\frac{5}{\pi }x_{1}‐6\right)}^{2}+10(1‐\frac{1}{8\pi })cosx_{1}+10$	2	[-5,10] × [0,15]	0.397887
$\begin{array}{l} F_{18}(x)=[1+(x_{1}+x_{2}+1{)}^{2}(19‐14x_{1}+3x_{1}^{2}‐14x_{2}+6x_{1}x_{2}+3x_{2}^{2})]\\ \;\times [30+(2x_{1}‐3x_{2}{)}^{2}(18‐32x_{1}+12x_{1}^{2}+48x_{2}‐36x_{1}x_{2}+27x_{2}^{2})] \end{array}$	2	[-2,2]^D^	3
$F_{19}(x)=‐\sum_{i=1}^{4}c_{i}exp(‐{\sum_{j=1}^{3}a_{ij}(x_{j}‐p_{ij})}^{2})$	3	[0,1]^D^	-3.86278
$F_{20}(x)=‐\sum_{i=1}^{4}c_{i}exp(‐{\sum_{j=1}^{6}a_{ij}(x_{j}‐p_{ij})}^{2})$	6	[0,1]^D^	-3.322
$F_{21}(x)=‐{\sum_{i=1}^{5}\left[(x‐a_{i}){\left(x‐a_{i}\right)}^{T}+c_{i}\right]}^{‐1}$	4	[0,10]^D^	-10.1532
$F_{22}(x)=‐{\sum_{i=1}^{7}\left[(x‐a_{i}){\left(x‐a_{i}\right)}^{T}+c_{i}\right]}^{‐1}$	4	[0,10]^D^	-10.40294
$F_{23}(x)=‐{\sum_{i=1}^{7}\left[(x‐a_{i}){\left(x‐a_{i}\right)}^{T}+c_{i}\right]}^{‐1}$	4	[0,10]^D^	-10.5364

This comprehensive set of benchmark functions allows for a thorough evaluation of MWOA across a spectrum of optimization challenges, ranging from unimodal to multimodal, and from single-objective to multi-objective scenarios. The results obtained from these experiments serve to provide valuable insights into the algorithm’s robustness and effectiveness across diverse problem domains.

### 4.1 Computational complexity of MWOA

MOWA is delineated by considering two critical aspects: time complexity and space complexity. These facets play pivotal roles in assessing the overall performance of an algorithm. Evaluating the time complexity provides insights into the algorithm’s efficiency in terms of execution speed, while analyzing space complexity helps gauge its efficiency in memory utilization. Both of these aspects are crucial metrics in determining the algorithm’s practicality and suitability for various computational tasks.

#### (1) Time complexity

MWOA’s performance is intricately affected by essential factors like the quantity of particles(*N*), the duration of iterations(*t*), and the expense associated with function evaluation(*c*). A comprehensive assessment of time complexity necessitates a thorough integration of their collective effects to derive an accurate evaluation. It is noteworthy that the time complexity of MWOA remains on par with that of the WOA, as indicated by the constancy maintained through Eqs (19) and (20). This observation underscores the stability of MWOA in terms of time complexity, emphasizing its efficiency in handling optimization tasks across various settings and computational scenarios.


O(WOA)=O(tNc) (19)

O(MWOA)=O(tNc) (20)

#### (2) Space complexity

In terms of space complexity, the consideration is focused solely on the initial stage—specifically, the entirety of the search space. In this context, the space complexity of MWOA is succinctly expressed as *O*(n). This notation signifies a linear relationship with the dimensionality of the problem, underscoring the algorithm’s ability to efficiently manage memory resources as it scales with the size of the optimization problem.

### 4.2 Comparison algorithm selection

To assess the fault detection classification capability of MWOA, it underwent comparison with several established nature-inspired metaheuristic algorithms. Table 5 presents the parameter settings for these comparison algorithms.

**Table 5:** The initial parameter settings for the corresponding algorithms.

Algorithms	Parameters	Values
WOA prototypes		
WOA	*a*(linear)	[0,2]
	*b*	1
WOA variants		
MWOA	*a*(nonlinear)	[0,2]
	*a*_*0*_	3
	*k*	5
	*a*_*min*_	0.0001
	*a*_*max*_	2
	*G*	1
	*α*	0.3
	***β***	0.001
Traditional and popular algorithms		
PSO	Coefficient of the cognitive component	2
	Coefficient of the social component	2
	[*V*_*max*_,*V*_*min*_]	[-2,2]
MPA	FADs	0.2
	Control step	0.5
GWO	*a*(linear)	[0,2]
	*r*	[0,1]
ABC	Upper limit of acceleration factor	1
	Number of reconnaissance bees	N
AOA	*α*	5
	*μ*	0.5

### 4.3 Sensitivity analysis of MWOA’s own parameter selection

At the beginning of this section, we tested the effect of varying the MWOA parameter values on its performance. Different scenarios were selected based on the values of the MWOA parameters (***k*** and ***α***). These parameters took values of 4, 5 and 6, respectively, so that we constructed 9 different scenarios (as shown in Table 6). Among the 23 benchmark functions used, we evaluated the performance of each scenario and collected the corresponding statistical results as detailed in Table 7.

**Table 6:** Scenarios of the tuning parameters.

Scenario No.	*k* value	*α* value
1	4	0.2
2	5	0.2
3	6	0.2
4	4	0.3
5	5	0.3
6	6	0.3
7	4	0.4
8	5	0.4
9	6	0.4

**Table 7:** The influence of the MWOA parameters (i.e., *k* and *α*) on CEC2005 functions.

Function		Scenario No.								
		Scenario 1	Scenario 2	Scenario 3	Scenario 4	Scenario 5	Scenario 6	Scenario 7	Scenario 8	Scenario 9
***F***_***1***_	Std	0.000E+00	0.000E+00	0.000E+00	0.000E+00	**0.000E+00**	0.000E+00	0.000E+00	0.000E+00	0.000E+00
	Mean	0.000E+00	0.000E+00	0.000E+00	0.000E+00	**0.000E+00**	0.000E+00	0.000E+00	0.000E+00	0.000E+00
	Best	0.000E+00	0.000E+00	0.000E+00	0.000E+00	**0.000E+00**	0.000E+00	0.000E+00	0.000E+00	0.000E+00
	Worst	0.000E+00	0.000E+00	0.000E+00	0.000E+00	**0.000E+00**	0.000E+00	0.000E+00	0.000E+00	0.000E+00
	Rank	1	1	1	1	**1**	1	1	1	1
***F***_***2***_	Std	0.000E+00	0.000E+00	0.000E+00	0.000E+00	**0.000E+00**	0.000E+00	0.000E+00	0.000E+00	0.000E+00
	Mean	0.000E+00	0.000E+00	0.000E+00	0.000E+00	**0.000E+00**	2.673E-323	3.597E-323	2.472E-322	1.851E-290
	Best	0.000E+00	0.000E+00	0.000E+00	0.000E+00	**0.000E+00**	2.862E-323	3.573E-323	2.478E-322	1.851E-290
	Worst	0.000E+00	0.000E+00	0.000E+00	0.000E+00	**0.000E+00**	1.247E-322	1.526E-322	2.962E-322	1.851E-289
	Rank	1	1	1	1	**1**	6	7	8	9
***F***_***3***_	Std	0.000E+00	0.000E+00	0.000E+00	0.000E+00	**0.000E+00**	0.000E+00	0.000E+00	0.000E+00	0.000E+00
	Mean	0.000E+00	0.000E+00	0.000E+00	0.000E+00	**0.000E+00**	0.000E+00	0.000E+00	0.000E+00	0.000E+00
	Best	0.000E+00	0.000E+00	0.000E+00	0.000E+00	**0.000E+00**	0.000E+00	0.000E+00	0.000E+00	0.000E+00
	Worst	0.000E+00	0.000E+00	0.000E+00	0.000E+00	**0.000E+00**	0.000E+00	0.000E+00	0.000E+00	0.000E+00
	Rank	1	1	1	1	**1**	1	1	1	1
***F***_***4***_	Std	0.000E+00	0.000E+00	0.000E+00	0.000E+00	**0.000E+00**	0.000E+00	0.000E+00	0.000E+00	0.000E+00
	Mean	0.000E+00	0.000E+00	0.000E+00	0.000E+00	**0.000E+00**	0.000E+00	4.927E-324	2.566E-295	4.506E-259
	Best	0.000E+00	0.000E+00	0.000E+00	0.000E+00	**0.000E+00**	0.000E+00	4.939E-324	1.873E-323	4.918E-278
	Worst	0.000E+00	0.000E+00	0.000E+00	0.000E+00	**0.000E+00**	4.981E-324	4.917E-324	2.566E-294	4.412E-258
	Rank	1	1	1	1	**1**	6	7	8	9
***F***_***5***_	Std	8.722E-04	9.238E-04	7.485E-04	3.828E-04	**3.550E-04**	1.286E-03	6.341E-04	3.309E-03	2.517E-03
	Mean	1.175E-03	5.879E-04	5.521E-04	3.114E-04	**2.295E-04**	1.037E-03	6.694E-04	2.973E-03	3.155E-03
	Best	1.836E-04	3.979E-07	1.736E-05	5.693E-07	**8.462E-07**	1.494E-05	4.468E-05	7.619E-05	1.196E-04
	Worst	2.598E-03	2.945E-03	2.567E-03	1.247E-03	**8.770E-04**	3.605E-03	1.679E-03	1.130E-02	7.041E-03
	Rank	7	4	3	2	**1**	6	5	8	9
***F***_***6***_	Std	3.423E-06	1.162E-05	1.779E-05	1.331E-05	**2.233E-06**	1.947E-06	3.619E-06	7.234E-06	6.628E-06
	Mean	3.004E-06	6.095E-06	8.174E-06	7.770E-06	**2.124E-06**	2.159E-06	3.956E-06	4.988E-06	8.100E-06
	Best	1.599E-07	3.784E-09	1.465E-08	7.679E-08	**1.221E-07**	2.460E-07	4.651E-08	3.161E-07	1.413E-06
	Worst	1.099E-05	3.837E-05	5.716E-05	4.353E-05	**7.582E-06**	6.115E-06	1.113E-05	2.522E-05	2.279E-05
	Rank	3	6	9	7	**1**	2	4	5	8
***F***_***7***_	Std	7.501E-06	1.875E-05	5.937E-06	7.473E-06	**5.982E-06**	7.806E-06	5.220E-06	1.041E-05	1.027E-05
	Mean	8.127E-06	1.905E-05	7.963E-06	8.464E-06	**7.070E-06**	7.400E-06	8.599E-06	1.016E-05	1.171E-05
	Best	1.744E-06	6.724E-08	3.168E-07	2.036E-07	**1.816E-07**	5.348E-07	8.602E-07	1.802E-07	1.152E-06
	Worst	2.548E-05	6.137E-05	2.152E-05	2.220E-05	**1.820E-05**	2.106E-05	1.351E-05	3.099E-05	3.300E-05
	Rank	4	9	3	5	**1**	2	6	7	8
***F***_***8***_	Std	1.163E-01	7.440E+01	1.635E-01	1.930E+02	**3.334E-02**	1.632E-01	7.338E-02	6.515E-02	1.143E-01
	Mean	-1.257E+04	-1.255E+04	-1.257E+04	-1.249E+04	**-1.257E+04**	-1.257E+04	-1.257E+04	-1.257E+04	-1.257E+04
	Best	-1.257E+04	-1.257E+04	-1.257E+04	-1.257E+04	**-1.257E+04**	-1.257E+04	-1.257E+04	-1.257E+04	-1.257E+04
	Worst	-1.257E+04	-1.233E+04	-1.257E+04	-1.198E+04	**-1.257E+04**	-1.257E+04	-1.257E+04	-1.257E+04	-1.257E+04
	Rank	4	8	7	9	**1**	6	3	2	5
***F***_***9***_	Std	0.000E+00	0.000E+00	0.000E+00	0.000E+00	**0.000E+00**	0.000E+00	0.000E+00	0.000E+00	0.000E+00
	Mean	0.000E+00	0.000E+00	0.000E+00	0.000E+00	**0.000E+00**	0.000E+00	0.000E+00	0.000E+00	0.000E+00
	Best	0.000E+00	0.000E+00	0.000E+00	0.000E+00	**0.000E+00**	0.000E+00	0.000E+00	0.000E+00	0.000E+00
	Worst	0.000E+00	0.000E+00	0.000E+00	0.000E+00	**0.000E+00**	0.000E+00	0.000E+00	0.000E+00	0.000E+00
	Rank	1	1	1	1	**1**	1	1	1	1
***F***_***10***_	Std	0.000E+00	0.000E+00	0.000E+00	0.000E+00	**0.000E+00**	0.000E+00	0.000E+00	0.000E+00	0.000E+00
	Mean	8.882E-16	8.882E-16	8.882E-16	8.882E-16	**8.882E-16**	8.882E-16	8.882E-16	8.882E-16	8.882E-16
	Best	8.882E-16	8.882E-16	8.882E-16	8.882E-16	**8.882E-16**	8.882E-16	8.882E-16	8.882E-16	8.882E-16
	Worst	8.882E-16	8.882E-16	8.882E-16	8.882E-16	**8.882E-16**	8.882E-16	8.882E-16	8.882E-16	8.882E-16
	Rank	1	1	1	1	**1**	1	1	1	1
***F***_***11***_	Std	0.000E+00	0.000E+00	0.000E+00	0.000E+00	**0.000E+00**	0.000E+00	0.000E+00	0.000E+00	0.000E+00
	Mean	0.000E+00	0.000E+00	0.000E+00	0.000E+00	**0.000E+00**	0.000E+00	0.000E+00	0.000E+00	0.000E+00
	Best	0.000E+00	0.000E+00	0.000E+00	0.000E+00	**0.000E+00**	0.000E+00	0.000E+00	0.000E+00	0.000E+00
	Worst	0.000E+00	0.000E+00	0.000E+00	0.000E+00	**0.000E+00**	0.000E+00	0.000E+00	0.000E+00	0.000E+00
	Rank	1	1	1	1	**1**	1	1	1	1
***F***_***12***_	Std	3.465E-07	1.190E-07	2.464E-07	1.172E-07	**1.492E-07**	1.776E-07	2.380E-07	1.187E-07	1.909E-07
	Mean	2.707E-07	1.403E-07	2.505E-07	1.014E-07	**8.134E-08**	1.115E-07	2.501E-07	1.358E-07	2.986E-07
	Best	1.075E-08	1.846E-08	2.149E-09	1.511E-09	**1.350E-11**	3.827E-09	3.022E-08	4.763E-09	3.822E-08
	Worst	1.163E-06	3.828E-07	8.612E-07	3.704E-07	**4.389E-07**	5.956E-07	7.462E-07	3.317E-07	5.965E-07
	Rank	8	5	7	2	**1**	3	6	4	9
***F***_***13***_	Std	1.139E-05	**1.597E-06**	1.042E-05	3.575E-06	2.894E-06	2.288E-06	2.466E-06	1.214E-05	2.108E-06
	Mean	4.223E-06	**1.810E-06**	7.978E-06	2.442E-06	2.130E-06	2.227E-06	2.089E-06	1.233E-05	3.300E-06
	Best	2.531E-11	**1.938E-07**	7.221E-08	8.669E-08	3.161E-07	4.586E-08	3.683E-08	5.378E-08	1.016E-07
	Worst	3.663E-05	**4.770E-06**	2.898E-05	9.345E-06	9.989E-06	6.720E-06	8.169E-06	3.266E-05	6.069E-06
	Rank	**7**	**1**	8	5	3	4	2	9	6
***F***_***14***_	Std	2.295E-09	3.144E-01	4.191E-01	8.223E-09	**1.487E-10**	2.160E-10	3.684E-10	1.233E-09	1.954E-10
	Mean	9.980E-01	1.097E+00	1.197E+00	9.980E-01	**9.980E-01**	9.980E-01	9.980E-01	9.980E-01	9.980E-01
	Best	9.980E-01	9.980E-01	9.980E-01	9.980E-01	**9.980E-01**	9.980E-01	9.980E-01	9.980E-01	9.980E-01
	Worst	9.980E-01	1.992E+00	1.992E+00	9.980E-01	**9.980E-01**	9.980E-01	9.980E-01	9.980E-01	9.980E-01
	Rank	6	8	9	7	**1**	3	4	5	2
***F***_***15***_	Std	2.453E-05	1.530E-05	3.216E-05	3.695E-05	**1.295E-05**	1.505E-05	1.926E-05	2.395E-05	1.532E-05
	Mean	3.356E-04	3.276E-04	3.477E-04	3.496E-04	**3.217E-04**	3.446E-04	3.379E-04	3.335E-04	3.396E-04
	Best	3.099E-04	3.120E-04	3.128E-04	3.100E-04	**3.081E-04**	3.167E-04	3.154E-04	3.102E-04	3.151E-04
	Worst	3.818E-04	3.586E-04	4.115E-04	4.140E-04	**3.455E-04**	3.686E-04	3.672E-04	3.815E-04	3.681E-04
	Rank	4	2	8	9	**1**	7	5	3	6
***F***_***16***_	Std	4.495E-03	7.147E-03	7.841E-07	5.245E-06	**1.327E-02**	7.902E-07	9.609E-03	1.766E-06	8.041E-03
	Mean	-1.001E+00	-1.002E+00	-1.000E+00	-1.000E+00	**-1.006E+00**	-1.000E+00	-1.003E+00	-1.000E+00	-1.003E+00
	Best	-1.014E+00	-1.023E+00	-1.000E+00	-1.000E+00	**-1.032E+00**	-1.000E+00	-1.030E+00	-1.000E+00	-1.025E+00
	Worst	-1.000E+00	-1.000E+00	-1.000E+00	-1.000E+00	**-1.000E+00**	-1.000E+00	-1.000E+00	-1.000E+00	-1.000E+00
	Rank	5	4	7	9	**1**	6	2	8	3
***F***_***17***_	Std	2.609E-04	2.361E-04	2.043E-04	8.506E-05	**1.453E-05**	1.029E-04	7.081E-05	6.132E-05	4.730E-05
	Mean	3.981E-01	3.981E-01	3.980E-01	3.980E-01	**3.979E-01**	3.979E-01	3.979E-01	3.979E-01	3.979E-01
	Best	3.979E-01	3.979E-01	3.979E-01	3.979E-01	**3.979E-01**	3.979E-01	3.979E-01	3.979E-01	3.979E-01
	Worst	3.986E-01	3.985E-01	3.986E-01	3.981E-01	**3.979E-01**	3.982E-01	3.981E-01	3.980E-01	3.980E-01
	Rank	9	8	7	6	**1**	5	2	3	4
***F***_***18***_	Std	2.264E-04	2.084E-04	2.148E-04	1.106E-04	**2.640E-05**	3.587E-04	8.395E-05	7.626E-05	6.216E-05
	Mean	3.000E+00	3.000E+00	3.000E+00	3.000E+00	**3.000E+00**	3.000E+00	3.000E+00	3.000E+00	3.000E+00
	Best	3.000E+00	3.000E+00	3.000E+00	3.000E+00	**3.000E+00**	3.000E+00	3.000E+00	3.000E+00	3.000E+00
	Worst	3.001E+00	3.001E+00	3.001E+00	3.000E+00	**3.000E+00**	3.001E+00	3.000E+00	3.000E+00	3.000E+00
	Rank	8	5	7	6	**1**	9	4	3	2
***F***_***19***_	Std	2.797E-02	7.036E-03	6.630E-03	3.746E-03	**2.459E-03**	3.727E-03	4.790E-03	6.037E-03	2.880E-03
	Mean	-3.846E+00	-3.855E+00	-3.856E+00	-3.859E+00	**-3.861E+00**	-3.860E+00	-3.860E+00	-3.859E+00	-3.860E+00
	Best	-3.862E+00	-3.862E+00	-3.862E+00	-3.863E+00	**-3.863E+00**	-3.862E+00	-3.863E+00	-3.863E+00	-3.863E+00
	Worst	-3.780E+00	-3.837E+00	-3.838E+00	-3.851E+00	**-3.855E+00**	-3.852E+00	-3.847E+00	-3.843E+00	-3.855E+00
	Rank	9	8	7	5	**1**	3	4	6	2
***F***_***20***_	Std	6.823E-02	1.013E-01	8.275E-02	9.705E-02	**8.557E-02**	8.894E-02	1.011E-01	8.553E-02	8.329E-02
	Mean	-3.149E+00	-3.160E+00	-3.189E+00	-3.193E+00	**-3.245E+00**	-3.187E+00	-3.226E+00	-3.226E+00	-3.237E+00
	Best	-3.267E+00	-3.303E+00	-3.299E+00	-3.305E+00	**-3.315E+00**	-3.312E+00	-3.320E+00	-3.322E+00	-3.319E+00
	Worst	-3.050E+00	-2.992E+00	-3.044E+00	-3.056E+00	**-3.025E+00**	-3.049E+00	-3.077E+00	-3.076E+00	-3.133E+00
	Rank	9	8	6	5	**1**	7	3	4	2
***F***_***21***_	Std	3.640E-03	4.655E-03	3.370E-03	1.188E-03	**3.263E-04**	8.624E-04	5.278E-04	2.827E-04	7.093E-04
	Mean	-1.015E+01	-1.015E+01	-1.015E+01	-1.015E+01	**-1.015E+01**	-1.015E+01	-1.015E+01	-1.015E+01	-1.015E+01
	Best	-1.015E+01	-1.015E+01	-1.015E+01	-1.015E+01	**-1.015E+01**	-1.015E+01	-1.015E+01	-1.015E+01	-1.015E+01
	Worst	-1.014E+01	-1.014E+01	-1.014E+01	-1.015E+01	**-1.015E+01**	-1.015E+01	-1.015E+01	-1.015E+01	-1.015E+01
	Rank	8	9	7	6	**1**	4	3	2	5
***F***_***22***_	Std	3.098E-02	9.970E-03	4.141E-03	5.831E-04	**2.646E-04**	2.884E-04	1.296E-03	2.710E-04	3.956E-04
	Mean	-1.039E+01	-1.040E+01	-1.040E+01	-1.040E+01	**-1.040E+01**	-1.040E+01	-1.040E+01	-1.040E+01	-1.040E+01
	Best	-1.040E+01	-1.040E+01	-1.040E+01	-1.040E+01	**-1.040E+01**	-1.040E+01	-1.040E+01	-1.040E+01	-1.040E+01
	Worst	-1.033E+01	-1.038E+01	-1.039E+01	-1.040E+01	**-1.040E+01**	-1.040E+01	-1.040E+01	-1.040E+01	-1.040E+01
	Rank	9	8	7	5	**1**	3	6	2	4
***F***_***23***_	Std	5.464E-02	3.961E-03	7.412E-04	5.125E-03	**4.137E-04**	1.405E-03	3.456E-04	1.138E-03	2.917E-04
	Mean	-1.052E+01	-1.053E+01	-1.054E+01	-1.053E+01	**-1.054E+01**	-1.053E+01	-1.054E+01	-1.054E+01	-1.054E+01
	Best	-1.054E+01	-1.054E+01	-1.054E+01	-1.054E+01	**-1.054E+01**	-1.054E+01	-1.054E+01	-1.054E+01	-1.054E+01
	Worst	-1.036E+01	-1.052E+01	-1.053E+01	-1.052E+01	**-1.054E+01**	-1.053E+01	-1.054E+01	-1.053E+01	-1.054E+01
	Rank	9	8	3	**7**	**1**	6	4	5	2
Mean Rank		5.04	4.69	4.87	4.43	**1.07**	4.04	3.57	4.09	4.34
Final Ranking		9	7	8	6	**1**	3	2	4	5

From these results, it can be seen that the fifth scenario (i.e., *α* = 0.3, ***k*** = 5) has the best performance among all the functions tested; it is closely followed by the seventh and the sixth scenarios, which obtained the second and third rankings, respectively. This suggests that the combination of ***α*** = 0.3 with different values of k has a significant effect on the optimization performance of the MWOA algorithm, especially the effect is most pronounced at ***k*** = 5.

Through this study, we are able to gain a deeper understanding of the impact of parameter settings on the performance of MWOA. Optimizing the parameter settings not only helps to improve the performance of MWOA on multiple benchmark functions, but also enhances its robustness and adaptability in practical applications.

### 4.4 Comparison of the MWOA with traditional algorithms

In the assessment of applicability and interpretability, a comparative analysis has been conducted among various optimization algorithms, including traditional and widely-used methods such as WOA, GWO [55], MPA [56], PSO [57], ABC [58], AOA [59] and SABO [60]. To ensure a fair competition between algorithms, each function was run independently for 50 times and the population size was set to ***N*** = 50. the number of iterations ***T*** = 1000 was used as the termination condition. Eventually, the best fitness value (Best), the worst fitness value (Worst), the average fitness value (Mean) and the standard deviation (Standard Deviation) of each algorithm in 50 runs are used as metrics for statistical analysis.The outcomes presented in Table 6 highlight the superior performance of the MWOA, as evidenced by its first average ranking and overall ranking among the considered algorithms.

**Fig 5:**
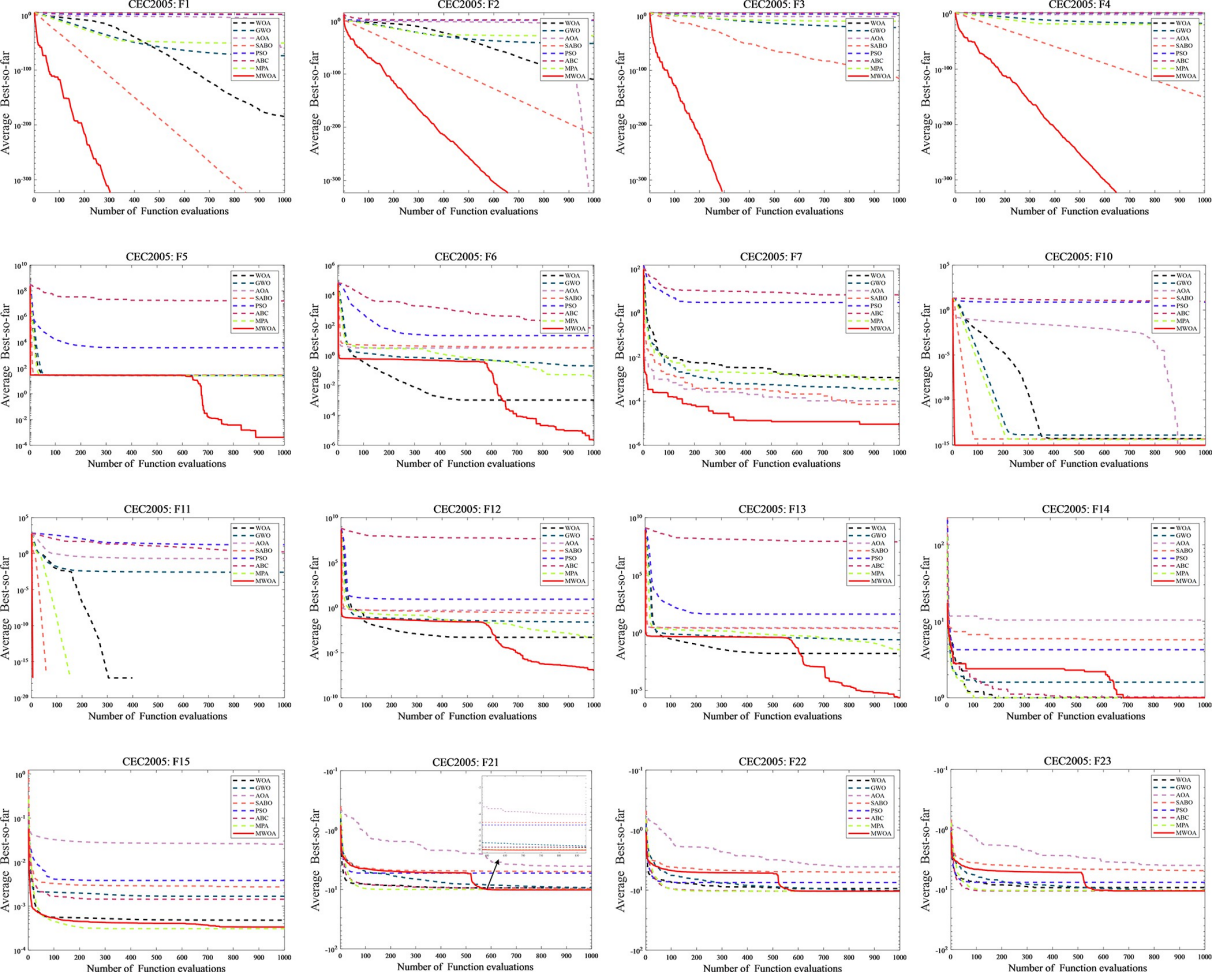
Convergence curve of MWOA and other traditional algorithms.

Table 8 results underscore the effectiveness of MWOA across a spectrum of optimization challenges, positioning it favorably when compared to other well-established algorithms. This success can be attributed to its unique characteristics and adaptability in navigating diverse solution spaces. Moreover, the convergence curves depicted in Fig 5 offer insights into the dynamic behavior of the MWOA in comparison to WOA, GWO, MPA, PSO, ABC, AOA and SABO algorithms. The curves illustrate that MWOA exhibits rapid convergence and adeptly avoids local stagnation. This characteristic is crucial in ensuring the algorithm’s efficiency in exploring the solution space and reaching optimal solutions in a timely manner.

**Table 8:** Experimental comparison of MWOA with other algorithms.

Function		Experimental Comparison							
		WOA	GWO	AOA	SABO	PSO	ABC	MPA	MWOA
***F***_***1***_	Std	0.000E+00	1.830E-73	1.113E-21	0.000E+00	4.921E+00	3.734E+01	3.005E-50	**0.000E+00**
	Mean	2.114E-174	5.151E-74	1.574E-22	0.000E+00	1.462E+01	4.924E+01	1.127E-50	**0.000E+00**
	Best	1.116E-192	5.337E-77	4.690E-165	0.000E+00	7.201E+00	6.650E+00	1.412E-54	**0.000E+00**
	Worst	1.041E-172	1.151E-72	7.871E-21	0.000E+00	3.093E+01	1.937E+02	1.813E-49	**0.000E+00**
***F***_***2***_	Std	1.927E-109	5.701E-43	0.000E+00	0.000E+00	4.147E+00	3.828E+01	1.312E-27	**0.000E+00**
	Mean	2.935E-110	4.616E-43	0.000E+00	4.579E-215	1.664E+01	4.349E+01	4.050E-28	**0.000E+00**
	Best	1.141E-118	7.449E-45	0.000E+00	2.067E-219	9.045E+00	1.999E-02	3.551E-31	**0.000E+00**
	Worst	1.363E-108	3.751E-42	0.000E+00	1.422E-213	2.398E+01	1.171E+02	8.618E-27	**0.000E+00**
***F***_***3***_	Std	5.898E+03	1.046E-20	1.027E-02	1.939E-114	3.765E+02	1.220E+04	1.591E-10	**0.000E+00**
	Mean	7.995E+03	1.587E-21	4.235E-03	3.587E-115	8.285E+02	7.183E+04	4.694E-11	**0.000E+00**
	Best	1.062E+02	2.907E-28	1.957E-130	2.424E-186	2.548E+02	4.838E+04	3.799E-17	**0.000E+00**
	Worst	2.102E+04	7.401E-20	5.090E-02	1.283E-113	1.743E+03	9.709E+04	9.877E-10	**0.000E+00**
***F***_***4***_	Std	2.698E+01	2.584E-18	2.034E-02	9.091E-151	2.666E+00	5.381E+00	1.416E-19	**0.000E+00**
	Mean	2.713E+01	1.685E-18	2.482E-02	2.906E-151	9.456E+00	6.586E+01	1.549E-19	**0.000E+00**
	Best	7.676E-10	2.656E-20	2.820E-91	1.849E-156	4.394E+00	5.356E+01	6.687E-21	**0.000E+00**
	Worst	8.699E+01	1.371E-17	4.697E-02	4.058E-150	1.673E+01	7.470E+01	7.018E-19	**0.000E+00**
***F***_***5***_	Std	3.073E-01	7.944E-01	2.557E-01	5.904E-01	2.920E+03	5.122E+06	7.526E-01	**6.383E-04**
	Mean	2.642E+01	2.642E+01	2.849E+01	2.826E+01	3.611E+03	1.223E+07	2.500E+01	**6.038E-04**
	Best	2.593E+01	2.495E+01	2.791E+01	2.701E+01	6.551E+02	3.230E+06	2.346E+01	**2.660E-05**
	Worst	2.698E+01	2.874E+01	2.891E+01	2.884E+01	1.330E+04	2.417E+07	2.708E+01	**2.463E-03**
***F***_***6***_	Std	9.174E-04	2.793E-01	2.967E-01	4.790E-01	5.845E+00	6.881E+01	1.633E-02	**7.301E-06**
	Mean	1.940E-03	3.397E-01	3.132E+00	2.504E+00	1.632E+01	4.716E+01	5.966E-03	**4.721E-06**
	Best	6.011E-04	4.855E-06	2.340E+00	1.718E+00	5.964E+00	5.306E+00	**9.898E-10**	3.567E-09
	Worst	4.849E-03	1.009E+00	3.931E+00	3.460E+00	3.221E+01	4.541E+02	8.196E-02	**3.170E-05**
***F***_***7***_	Std	1.012E-03	2.500E-04	7.887E-05	7.653E-05	3.627E+00	1.995E+00	4.887E-04	**1.054E-05**
	Mean	8.811E-04	4.194E-04	7.240E-05	9.587E-05	1.858E+00	6.532E+00	7.409E-04	**1.236E-05**
	Best	3.486E-06	7.309E-05	1.489E-06	2.882E-06	4.523E-02	2.655E+00	1.610E-04	**2.138E-07**
	Worst	3.716E-03	1.257E-03	3.842E-04	2.997E-04	2.175E+01	1.063E+01	2.048E-03	**4.032E-05**
***F***_***8***_	Std	1.090E+03	9.012E+02	4.387E+02	2.991E+02	4.632E+02	1.404E+117	4.096E+02	**6.395E+01**
	Mean	-1.183E+04	-6.200E+03	-5.270E+03	-3.009E+03	-2.746E+03	-2.626E+116	-9.222E+03	**-1.256E+04**
	Best	-1.257E+04	-7.685E+03	-6.378E+03	-3.748E+03	-3.901E+03	-9.207E+117	-1.010E+04	**-1.257E+04**
	Worst	-8.837E+03	-3.429E+03	-4.515E+03	-2.535E+03	-1.842E+03	-2.782E+105	-8.292E+03	**-1.212E+04**
***F***_***9***_	Std	0.000E+00	8.461E-01	0.000E+00	0.000E+00	2.571E+01	1.769E+01	0.000E+00	**0.000E+00**
	Mean	0.000E+00	1.197E-01	0.000E+00	0.000E+00	1.317E+02	2.629E+02	0.000E+00	**0.000E+00**
	Best	0.000E+00	0.000E+00	0.000E+00	0.000E+00	7.359E+01	2.186E+02	0.000E+00	**0.000E+00**
	Worst	0.000E+00	5.983E+00	0.000E+00	0.000E+00	2.127E+02	2.942E+02	0.000E+00	**0.000E+00**
***F***_***10***_	Std	2.412E-15	2.803E-15	0.000E+00	5.024E-16	1.378E+00	1.869E+00	1.245E-15	**0.000E+00**
	Mean	3.659E-15	1.261E-14	8.882E-16	4.512E-15	7.036E+00	8.665E+00	3.944E-15	**8.882E-16**
	Best	8.882E-16	7.994E-15	8.882E-16	4.441E-15	4.877E+00	4.722E+00	8.882E-16	**8.882E-16**
	Worst	7.994E-15	1.510E-14	8.882E-16	7.994E-15	1.002E+01	1.275E+01	4.441E-15	**8.882E-16**
***F***_***11***_	Std	1.401E-02	5.132E-03	1.308E-01	0.000E+00	7.727E+00	1.257E+00	0.000E+00	**0.000E+00**
	Mean	4.336E-03	2.173E-03	1.807E-01	0.000E+00	1.340E+01	1.507E+00	0.000E+00	**0.000E+00**
	Best	0.000E+00	0.000E+00	1.523E-03	0.000E+00	4.127E+00	1.017E+00	0.000E+00	**0.000E+00**
	Worst	7.148E-02	1.769E-02	4.959E-01	0.000E+00	3.733E+01	9.762E+00	0.000E+00	**0.000E+00**
***F***_***12***_	Std	3.941E-03	9.653E-03	4.986E-02	1.185E-01	3.432E+00	1.411E+07	4.255E-04	**4.539E-07**
	Mean	1.229E-03	1.893E-02	5.062E-01	2.205E-01	8.619E+00	2.987E+07	1.602E-04	**2.520E-07**
	Best	9.808E-05	6.378E-03	3.797E-01	7.791E-02	3.113E+00	4.155E+06	3.408E-10	**6.430E-11**
	Worst	2.559E-02	3.946E-02	6.473E-01	8.106E-01	1.803E+01	5.854E+07	2.045E-03	**2.422E-06**
***F***_***13***_	Std	5.988E-02	1.691E-01	1.174E-01	4.157E-01	2.656E+01	2.720E+07	1.650E-01	**5.876E-06**
	Mean	2.101E-02	2.509E-01	2.812E+00	2.744E+00	4.375E+01	6.337E+07	7.569E-02	**3.545E-06**
	Best	1.450E-03	7.557E-06	2.485E+00	1.604E+00	6.785E+00	1.877E+07	4.704E-09	**2.001E-08**
	Worst	4.286E-01	8.125E-01	2.994E+00	3.015E+00	1.713E+02	1.624E+08	1.144E+00	**2.801E-05**
***F***_***14***_	Std	1.482E+00	3.173E+00	4.040E+00	3.885E+00	3.053E+00	9.792E-03	**3.172E-17**	1.885E-05
	Mean	1.431E+00	2.962E+00	9.618E+00	5.485E+00	3.250E+00	1.001E+00	**9.980E-01**	9.980E-01
	Best	9.980E-01	9.980E-01	9.980E-01	9.994E-01	9.980E-01	9.980E-01	**9.980E-01**	9.980E-01
	Worst	1.076E+01	1.267E+01	1.267E+01	1.279E+01	1.737E+01	1.063E+00	**9.980E-01**	9.981E-01
***F***_***15***_	Std	4.170E-04	6.584E-03	2.949E-02	3.257E-03	5.405E-03	3.005E-04	**3.030E-19**	3.299E-05
	Mean	7.046E-04	2.714E-03	1.500E-02	1.448E-03	2.406E-03	1.375E-03	**3.075E-04**	3.447E-04
	Best	3.076E-04	3.075E-04	3.306E-04	3.158E-04	3.075E-04	1.043E-03	**3.075E-04**	3.079E-04
	Worst	2.237E-03	2.036E-02	1.184E-01	1.932E-02	2.036E-02	2.536E-03	**3.075E-04**	4.297E-04
***F***_***16***_	Std	3.998E-12	2.737E-09	1.237E-07	1.032E-02	2.220E-16	2.555E-05	**1.765E-16**	1.702E-06
	Mean	-1.032E+00	-1.032E+00	-1.032E+00	-1.025E+00	-1.032E+00	-1.032E+00	**-1.032E+00**	-1.000E+00
	Best	-1.032E+00	-1.032E+00	-1.032E+00	-1.032E+00	-1.032E+00	-1.032E+00	**-1.032E+00**	-1.000E+00
	Worst	-1.032E+00	-1.032E+00	-1.032E+00	-9.974E-01	-1.032E+00	-1.032E+00	**-1.032E+00**	-1.000E+00
***F***_***17***_	Std	7.075E-08	8.294E-06	1.305E-02	1.288E-01	3.364E-16	3.282E-04	**3.364E-16**	3.936E-03
	Mean	**3.979E-01**	3.979E-01	4.114E-01	4.590E-01	3.979E-01	3.981E-01	3.979E-01	3.985E-01
	Best	3.979E-01	3.979E-01	3.984E-01	3.979E-01	3.979E-01	3.979E-01	3.979E-01	**3.979E-01**
	Worst	**3.979E-01**	3.979E-01	4.656E-01	1.042E+00	3.979E-01	3.993E-01	3.979E-01	4.258E-01
***F***_***18***_	Std	1.867E-06	2.136E-06	1.743E+01	4.849E+00	1.361E-15	7.692E-04	**1.409E-15**	2.885E-04
	Mean	3.000E+00	3.000E+00	1.342E+01	4.898E+00	3.000E+00	3.001E+00	3.000E+00	**3.000E+00**
	Best	3.000E+00	3.000E+00	3.000E+00	3.000E+00	3.000E+00	3.000E+00	3.000E+00	**3.000E+00**
	Worst	3.000E+00	3.000E+00	9.748E+01	2.532E+01	3.000E+00	3.004E+00	**3.000E+00**	3.001E+00
***F***_***19***_	Std	1.967E-03	2.553E-03	3.247E-03	3.668E-01	3.771E-03	7.550E-07	**3.132E-15**	6.919E-03
	Mean	-3.862E+00	-3.862E+00	-3.852E+00	-3.592E+00	-3.860E+00	-3.863E+00	**-3.863E+00**	-3.858E+00
	Best	-3.863E+00	-3.863E+00	-3.856E+00	-3.863E+00	-3.863E+00	-3.863E+00	-3.863E+00	**-3.863E+00**
	Worst	-3.855E+00	-3.855E+00	-3.840E+00	-1.946E+00	-3.855E+00	-3.863E+00	**-3.863E+00**	-3.821E+00
***F***_***20***_	Std	7.626E-02	8.042E-02	8.146E-02	1.380E-01	4.765E-01	**3.612E-05**	0.000E+00	9.502E-02
	Mean	-3.240E+00	-3.251E+00	-3.048E+00	-3.260E+00	-3.001E+00	**-3.322E+00**	-3.322E+00	-3.177E+00
	Best	-3.322E+00	-3.322E+00	-3.244E+00	-3.322E+00	-3.322E+00	**-3.322E+00**	-3.322E+00	-3.314E+00
	Worst	-3.012E+00	-3.133E+00	-2.815E+00	-2.643E+00	-1.070E+00	**-3.322E+00**	-3.322E+00	-2.850E+00
***F***_***21***_	Std	1.720E+00	1.539E+00	8.046E-01	1.334E+00	3.565E+00	1.040E+00	7.210E-01	**7.333E-04**
	Mean	-9.594E+00	-9.645E+00	-3.606E+00	-5.263E+00	-6.701E+00	-9.929E+00	-1.005E+01	**-1.015E+01**
	Best	-1.015E+01	-1.015E+01	-5.392E+00	-1.008E+01	-1.015E+01	-1.015E+01	-1.015E+01	**-1.015E+01**
	Worst	-2.630E+00	-5.055E+00	-2.181E+00	-1.768E+00	-2.630E+00	-3.418E+00	-5.055E+00	**-1.015E+01**
***F***_***22***_	Std	2.640E+00	1.052E+00	1.448E+00	1.009E+00	3.526E+00	3.485E-04	7.298E-15	**1.321E-03**
	Mean	-8.960E+00	-1.019E+01	-4.060E+00	-5.201E+00	-8.034E+00	-1.040E+01	-1.040E+01	**-1.040E+01**
	Best	-1.040E+01	-1.040E+01	-8.301E+00	-9.718E+00	-1.040E+01	-1.040E+01	-1.040E+01	**-1.040E+01**
	Worst	-2.766E+00	-5.088E+00	-1.634E+00	-3.694E+00	-1.838E+00	-1.040E+01	-1.040E+01	**-1.040E+01**
***F***_***23***_	Std	1.672E+00	7.582E-01	1.272E+00	2.102E+00	3.826E+00	5.404E-04	1.297E+00	**8.381E-04**
	Mean	-1.002E+01	-1.043E+01	-4.005E+00	-5.559E+00	-7.586E+00	-1.054E+01	-1.021E+01	**-1.054E+01**
	Best	-1.054E+01	-1.054E+01	-7.340E+00	-1.040E+01	-1.054E+01	-1.054E+01	-1.054E+01	**-1.054E+01**
	Worst	-2.807E+00	-5.175E+00	-1.758E+00	-2.600E+00	-2.422E+00	-1.053E+01	-5.128E+00	**-1.053E+01**

**Fig 6:**
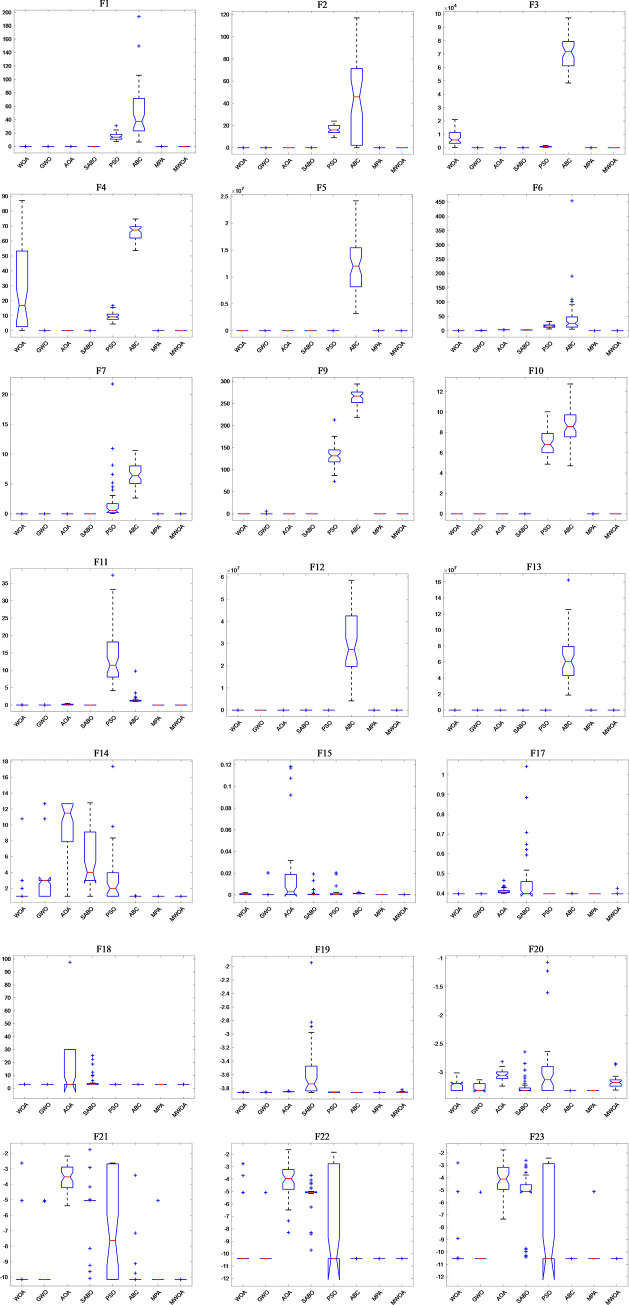
Boxplots of TTAO and 7 comparison algorithms on CEC2005 functions.

In order to further verify the optimization stability of the MWOA algorithm, the boxplots of each algorithm on the CEC2005 benchmark function are recorded in Fig 6. It is obvious from the figure that the results of the MWOA algorithm exhibit smaller upper and lower bound gaps among the 50 independent experiments, which indicates that the algorithm’s results have high consistency and stability across different runs. In contrast, the other algorithms have significantly larger upper and lower bound gaps, indicating that the results of these algorithms fluctuate more across runs. In addition, the MWOA algorithm also outperforms the other algorithms in all experiments in terms of worst-case results, showing its reliability and robustness in dealing with complex optimization problems.

The overall findings suggest that MWOA stands out as a promising optimization algorithm, showcasing competitive performance against established counterparts and demonstrating its potential for practical applications across various domains.

### 4.5 Statistical tests

Comparisons between algorithms do not fully guarantee the superiority and effectiveness of the algorithms due to the chance nature of the test results. Therefore, this subsection uses various statistical tests to demonstrate the statistical superiority of MWOA. Specifically, we performed statistical tests on the algorithm’s results on the CEC2005 function. In order to verify the significant difference between the MWOA algorithm and other comparative algorithms, the Wilcoxon rank sum test [61] was used for nonparametric testing. At the 5% significance level, if the p-value is less than 0.05, it means that the two algorithms are significantly different in a function, otherwise the difference is not significant.

First, we used MWOA as a control algorithm for pairwise comparison with other algorithms to generate p-values. Table 9 shows the statistical test results of Mann-Whitney U-test for the MWOA algorithm proposed in this paper at 5% significance level. In this table, “+” indicates that the algorithm has a significant advantage and “-” indicates that the algorithm is not significantly competitive in terms of statistical significance. Out of the 161 comparison tests conducted, 154 showed a significant advantage.The results of the Wilcoxon rank sum test clearly indicate that the MWOA algorithm outperforms the other compared algorithms.

**Table 9:** Wilcoxon rank sum test results from MWOA and 7 comparison algorithms on CEC2005 functions.

	MWOA vs. WOA	MWOA vs. GWO	MWOA vs. AOA	MWOA vs. SABO	MWOA vs. PSO	MWOA vs. ABC	MWOA vs. MPA
F1	1.464E-03 **(+)**	2.528E-03 **(+)**	1.430E-03 **(+)**	3.898E-03 **(+)**	6.389E-04 **(+)**	1.008E-05 **(+)**	5.230E-03 **(+)**
F2	1.464E-03 **(+)**	2.528E-03 **(+)**	1.430E-03 **(+)**	3.898E-03 **(+)**	6.389E-04 **(+)**	1.008E-05 **(+)**	5.230E-03 **(+)**
F3	1.464E-03 **(+)**	2.528E-03 **(+)**	1.430E-03 **(+)**	3.898E-03 **(+)**	6.389E-04 **(+)**	1.822E-05 **(+)**	5.230E-03 **(+)**
F4	1.464E-03 **(+)**	2.528E-03 **(+)**	1.430E-03 **(+)**	3.898E-03 **(+)**	6.389E-04 **(+)**	1.008E-05 **(+)**	5.230E-03 **(+)**
F5	1.464E-03 **(+)**	2.528E-03 **(+)**	1.430E-03 **(+)**	3.898E-03 **(+)**	6.389E-04 **(+)**	1.008E-05 **(+)**	5.230E-03 **(+)**
F6	1.464E-03 **(+)**	7.335E-03 **(+)**	1.430E-03 **(+)**	3.898E-03 **(+)**	6.389E-04 **(+)**	1.008E-05 **(+)**	5.230E-03 **(+)**
F7	1.464E-03 **(+)**	2.528E-03 **(+)**	2.673E-03 **(+)**	4.733E-03 **(+)**	6.389E-04 **(+)**	1.008E-05 **(+)**	5.230E-03 **(+)**
F8	2.754E-01 **(-)**	2.528E-03 **(+)**	1.430E-03 **(+)**	3.898E-03 **(+)**	6.389E-04 **(+)**	1.008E-05 **(+)**	1.385E-01 **(-)**
F9	1.464E-03 **(+)**	2.528E-03 **(+)**	1.430E-03 **(+)**	3.898E-03 **(+)**	6.389E-04 **(+)**	1.008E-05 **(+)**	5.230E-03 **(+)**
F10	1.464E-03 **(+)**	2.528E-03 **(+)**	1.430E-03 **(+)**	3.898E-03 **(+)**	7.663E-04 **(+)**	1.008E-05 **(+)**	5.230E-03 **(+)**
F11	1.464E-03 **(+)**	2.528E-03 **(+)**	1.430E-03 **(+)**	3.898E-03 **(+)**	6.389E-04 **(+)**	1.008E-05 **(+)**	5.230E-03 **(+)**
F12	1.464E-03 **(+)**	2.528E-03 **(+)**	1.430E-03 **(+)**	3.898E-03 **(+)**	6.389E-04 **(+)**	1.008E-05 **(+)**	5.230E-03 **(+)**
F13	1.464E-03 **(+)**	2.528E-03 **(+)**	1.430E-03 **(+)**	3.898E-03 **(+)**	6.389E-04 **(+)**	1.008E-05 **(+)**	5.740E-02 **(-)**
F14	7.476E-03 **(+)**	2.528E-03 **(+)**	1.430E-03 **(+)**	3.898E-03 **(+)**	6.389E-04 **(+)**	1.008E-05 **(+)**	5.230E-03 **(+)**
F15	1.464E-03 **(+)**	1.270E-01 **(-)**	1.430E-03 **(+)**	3.898E-03 **(+)**	6.389E-04 **(+)**	1.008E-05 **(+)**	5.138E-01 **(-)**
F16	1.464E-03 **(+)**	2.528E-03**(+)**	1.430E-03 **(+)**	3.898E-03 **(+)**	6.389E-04 **(+)**	1.008E-05 **(+)**	5.230E-03 **(+)**
F17	1.464E-03 **(+)**	2.528E-03 **(+)**	1.430E-03 **(+)**	1.679E-01 **(-)**	6.389E-04 **(+)**	1.008E-05 **(+)**	5.230E-03 **(+)**
F18	1.464E-03 **(+)**	2.528E-03 **(+)**	7.739E-03 **(+)**	3.898E-03 **(+)**	6.389E-04 **(+)**	1.008E-05 **(+)**	5.230E-03 **(+)**
F19	1.464E-03 **(+)**	2.528E-03 **(+)**	1.430E-03 **(+)**	3.898E-03 **(+)**	6.389E-04 **(+)**	1.008E-05 **(+)**	5.230E-03 **(+)**
F20	1.464E-03 **(+)**	2.528E-03 **(+)**	1.430E-03 **(+)**	3.898E-03 **(+)**	6.389E-04 **(+)**	1.008E-05 **(+)**	2.177E-01 **(-)**
F21	5.574E-03 **(+)**	2.528E-03 **(+)**	1.430E-03 **(+)**	3.898E-03 **(+)**	6.389E-04 **(+)**	7.146E-06 **(+)**	5.230E-03 **(+)**
F22	1.464E-03 **(+)**	2.528E-03 **(+)**	1.430E-03 **(+)**	3.898E-03 **(+)**	5.478E-04 **(+)**	1.008E-05 **(+)**	5.230E-03 **(+)**
F23	1.464E-03 **(+)**	2.528E-03 **(+)**	1.430E-03 **(+)**	3.898E-03 **(+)**	6.389E-04 **(+)**	1.008E-05 **(+)**	5.230E-03 **(+)**
**+/-.**	22/1	22/1	23/0	22/1	23/0	23/0	19/4

In summary, despite some randomness and uncertainty in algorithm comparison, the results of Wilcoxon rank sum test can prove that the MWOA algorithm is statistically significantly superior. This further validates the effectiveness and competitiveness of MWOA in solving the CEC2005 function problem.

## 5. MWOA-BiLSTM diagnostic model

**Fig 7:**
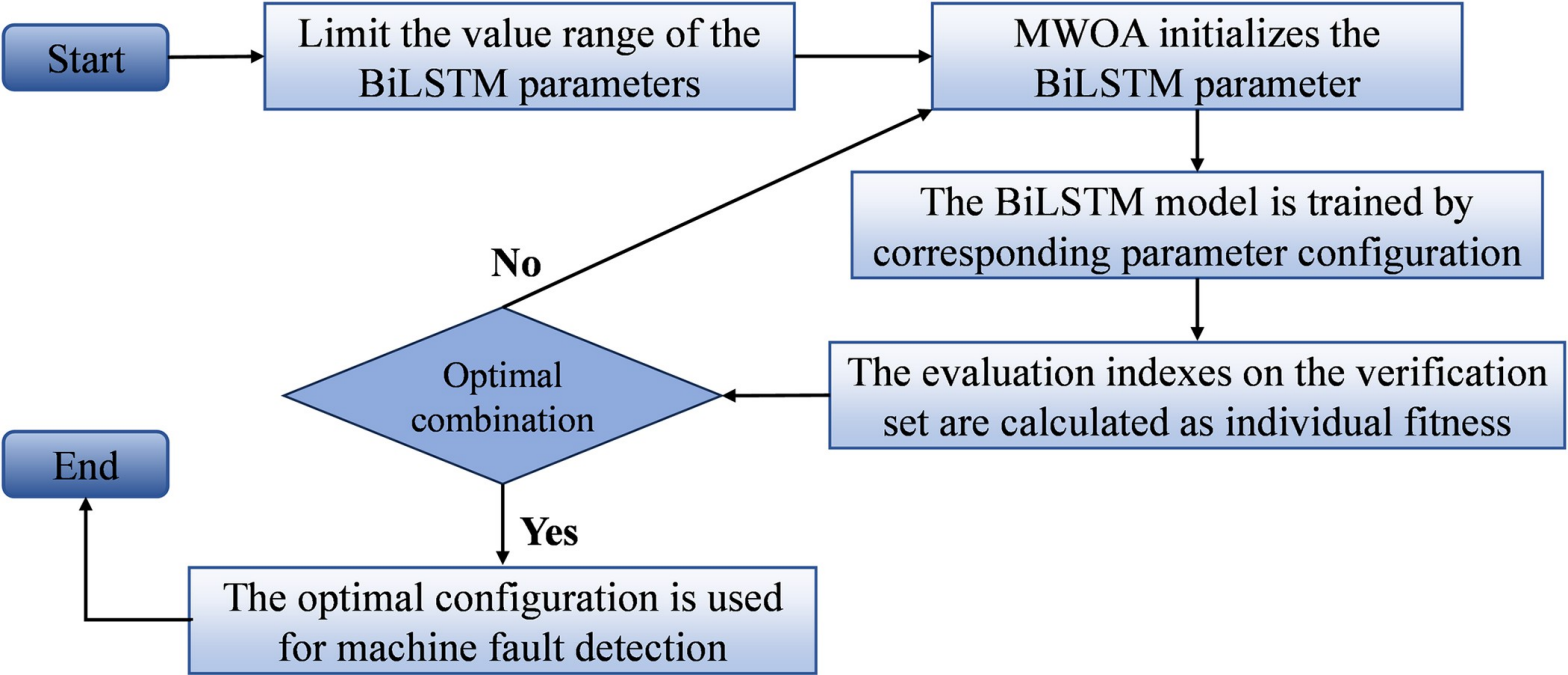
MWOA-BiLSTM machine fault detection process.

This study utilizes the MWOA to investigate the optimization performance of machine fault detection within industrial contexts. The algorithm iteratively improves a multilayer perceptron model by updating the number of hidden layer nodes, learning rate, and regularization parameters of the BiLSTM. Consequently, this process constructs a robust machine fault detection model, continuously refining these parameters for optimal performance. Through this approach, the study enhances the effectiveness of the BiLSTM model, resulting in significant improvements in classification rates and notable reductions in error rates. The MWOA-BiLSTM machine fault detection process is shown in Fig 7.

### 5.1 Machine predictive maintenance classification dataset

This paper uses the Machine Predictive Maintenance Classification Dataset from the University of California (UCI). The AI4I 2020 Predictive Maintenance Dataset is a synthetic collection designed to mirror real-world predictive maintenance data commonly found in industrial settings. It comprises 10,000 data points organized as rows, each containing 14 features across various columns. The Predictive Maintenance Dataset, comprising the five attributes illustrated in Table 10, is employed to identify the type of machine failure and validate the accuracy of the model. Furthermore, the dataset underwent partitioning into training and testing subsets, with a distribution ratio of 80% for training and 20% for testing.

**Table 10:** The attribution of the predictive maintenance dataset.

Attribution	Means
UID	unique identifier ranging from 1 to 10000
productID	“L”, “M” or“L” representing low (50%), medium (30%), and high (20%)
air temperature	normalized to a standard deviation of 2 K around 300 K
process temperature	generated using a random walk process normalized to a standard deviation of 1 K
rotational speed	calculated from powepower of 2860 W
torque	torque values are normally distributed around 40 Nm with an Ïƒ = 10 Nm
tool wear	H/M/L add 5/3/2 minutes of tool wear to the used tool
Failure	Power Failure
	Tool Wear Failure
	Overstrin Failure
	Random Failure
	Heat Dissipation Failure

#### 5.1.1 Dataset visualization

**Fig 8:**
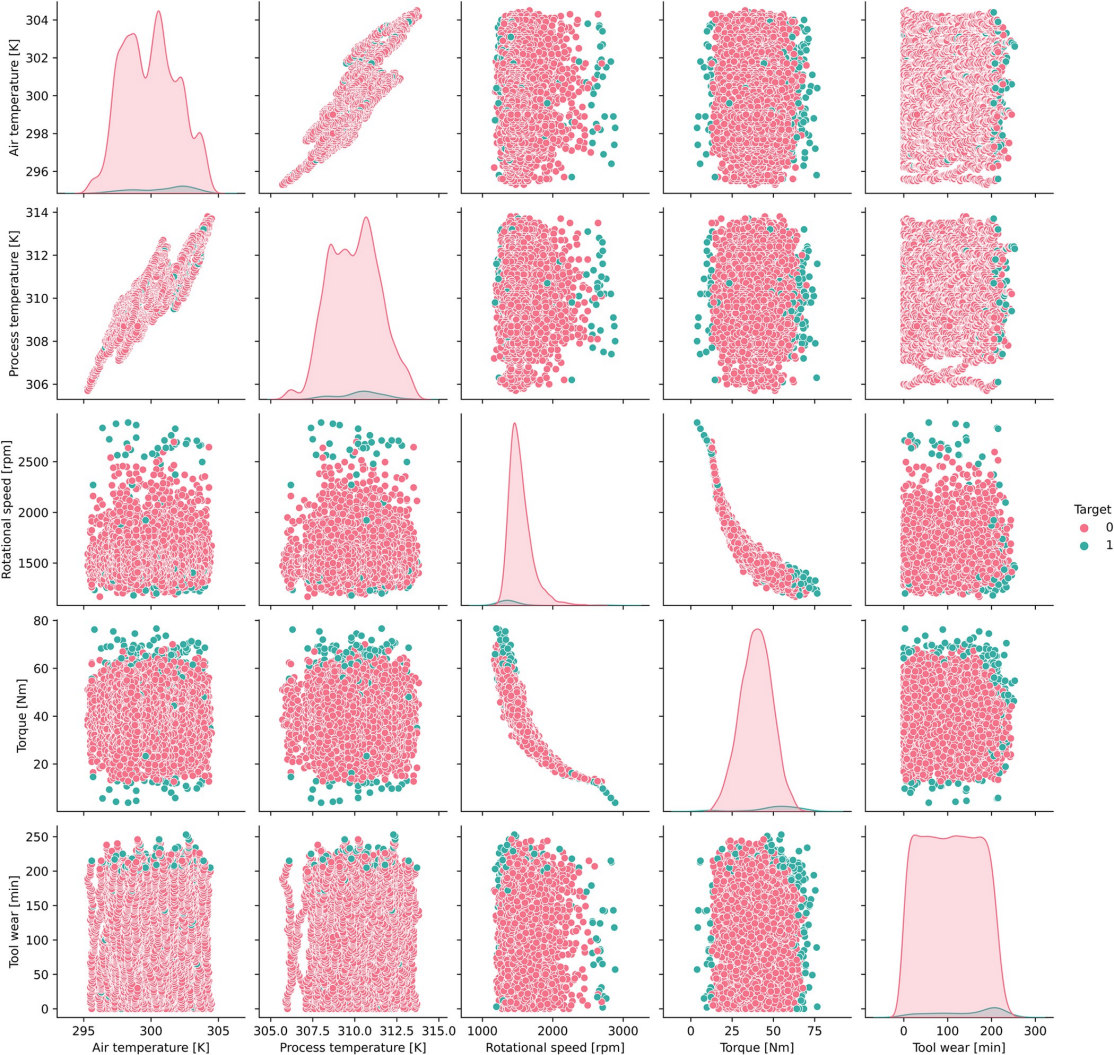
Fault interval.

By assigning the faulty and non-faulty cases in The AI4I 2020 Predictive Maintenance Dataset as Target "1" and "0" respectively, one can gain insight into the distribution of faulty data. This distribution is depicted in Fig 8, offering a visual portrayal for better comprehension.

**Fig 9:**
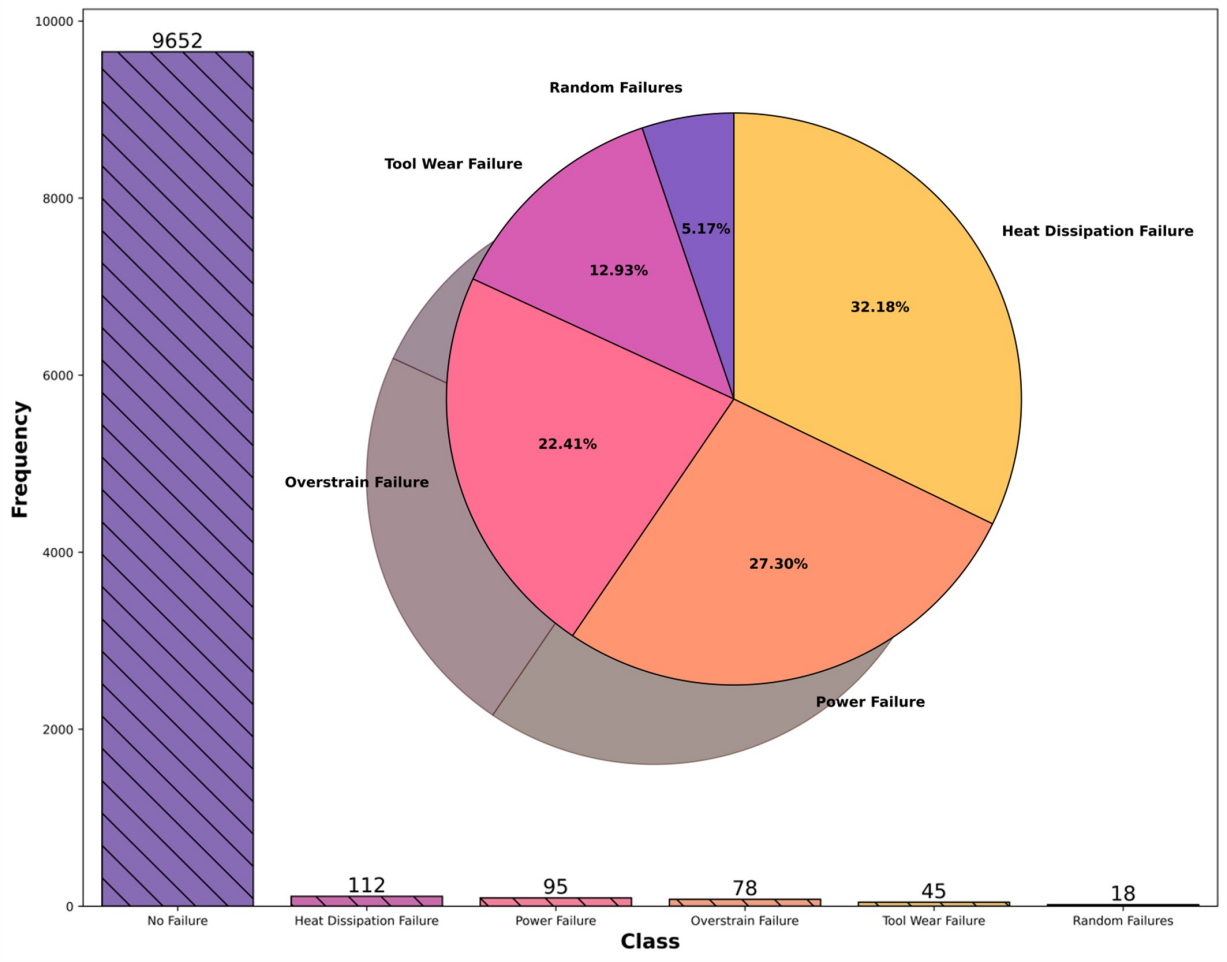
Fault classification proportion.

Among the 10000 data points, 9652 indicate a non-failure condition, while 348 denote a failure condition. These failures are categorized into five distinct situations based on various metrics: Power Failure, Tool Wear Failure, Overstrin Failure, Random Failure, and Heat Dissipation Failure. Detailed distributions are illustrated in Fig 9.

### 5.2 MWOA optimization of BiLSTM hyperparameters

In this paper, MWOA is integrated with the BiLSTM classifier to optimize its hyperparameters, including the learning rate, the number of hidden layer nodes, and the regularization coefficient. The goal is to maximize the classifier’s performance across four metrics: accuracy, precision, recall, and F1-Score. The learning rate, which controls the step size for updating network weights, is typically tuned within the range of [10^−6^,10^−1^]. The number of hidden layer nodes is the number of nodes in BiLSTM layer, and its value range is [10,30], while the regularization coefficient addresses overfitting and is adjusted within the range of [10^−5^,10^−1^]. The optimization problem is three-dimensional, as it involves optimizing three independent hyperparameters.

### 5.3 Experimental analysis

To validate the accuracy of the proposed MWOA-BiLSTM model in machine fault detection, This paper have assembled a variety of models for comparison, including WOA-BiLSTM, ABC-BiLSTM, BOA-BiLSTM, PSO-BiLSTM, SSA-BiLSTM, and MPA-BiLSTM. The evaluation of the MWOA-BiLSTM model’s performance is based on criteria such as accuracy, precision of the final classification, recall, and F1-Score. These metrics play a crucial role in evaluating the efficiency and dependability of the MWOA-BiLSTM model in identifying machine faults.

**Fig 10:**
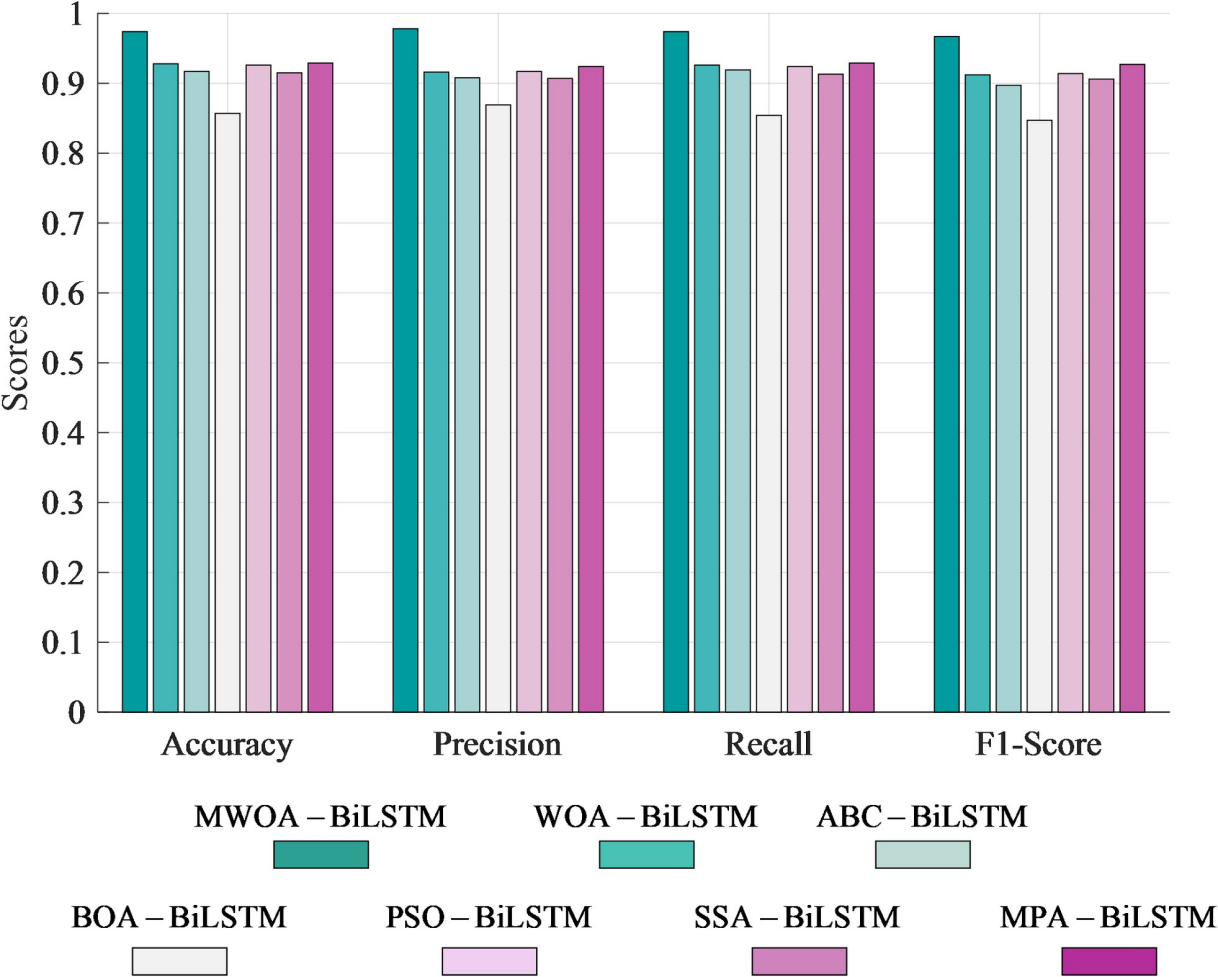
Comparison of evaluation indicators of different metaheuristic algorithm classifiers.

It’s evident from Fig 10 that the MWOA-BiLSTM model achieves a higher final classification rate compared to other methods. This underscores the excellent exploratory capability of the MWOA and its effectiveness in mitigating local optimal solutions in machine fault identification tasks. Notably, the MWOA-BiLSTM model demonstrates outstanding accuracy in recognizing machine faults, a critical aspect in industrial settings. The model surpasses others in key metrics such as accuracy, precision, recall, and F1-Score, showcasing its superior performance in tackling the challenges of machine fault identification. This outcome further confirms the robustness and efficiency of the MWOA within the search space. Therefore, the application of the MWOA-BiLSTM model in the industrial field holds significant importance in helping predict and maintain equipment faults more efficiently, thereby increasing productivity and reducing costs. To sum up, the incorporation of the MWOA algorithm represents a substantial improvement in both the classification rate and overall performance of machine fault recognition models. During the training process, the incorporation of a multilayer perceptron endows MWOA with potent exploratory capability, mitigates the risk of falling into local optimal solutions through adaptive improvement of the learning rate and regularization parameter, promotes effective updating of the weights and biases of the BiLSTM layer, and thus improves the classification rate.

## 6. Conclusion

This study systematically scrutinizes the performance of the newly developed metaheuristic algorithm, MWOA, through a comprehensive comparative analysis with other state-of-the-art meta-heuristics and proto-algorithms, including WOA, GWO, MPA, PSO, ABC, AOA and SABO. The evaluation of MWOA’s optimization capabilities was conducted using the CEC2005 benchmark functions and the AI4I 2020 Predictive Maintenance Dataset. Additionally, we conducted a comprehensive comparative analysis of MWOA-BiLSTM against its competitors. The key findings from this investigation are summarized below.

1. Across various functions, MWOA consistently outperforms the comparison algorithm.
2. MWOA exhibits faster convergence compared to the comparative algorithm across a wide range of functions, particularly evident in functions F1~5, F7~13, F15, and F21~F23.
3. MWOA offers notable advantages in terms of computational cost and complexity, ensuring optimal results.
4. MWOA was verified to be significantly different from other comparative algorithms by the Wilcoxon rank sum test
5. The performance of MWOA-BiLSTM on The AI4I 2020 Predictive Maintenance Dataset, including metrics such as accuracy, precision, recall, and F1-Score, significantly surpasses that of WOA-BiLSTM, ABC-BiLSTM, BOA-BiLSTM, PSO-BiLSTM, SSA-BiLSTM, and MPA-BiLSTM.

Inspired by MWOA, in the realm of optimization strategy design, researchers can contemplate the integration of different metaheuristics to leverage synergistic advantages. This entails the simultaneous application of multiple metaheuristic algorithms in problem-solving endeavors, harnessing their respective strengths across various stages or contexts. Such an amalgamative approach holds the promise of enhancing optimization performance and fortifying algorithms with heightened resilience and adaptability.
